# Experimental validation of an adaptive fuzzy logic controller for MPPT of grid connected PV system

**DOI:** 10.1038/s41598-025-10188-7

**Published:** 2025-07-25

**Authors:** Basem E. Elnaghi, Ahmed M. Ismaiel, Mohamed M. Ismail, Honey A. Zedan, Ahmed A. Salem

**Affiliations:** 1 Electrical power and machines department, Faculty of Engineering, Suez Canal University, Ismailia, 41522 Egypt; 2 Electrical engineering department, Faculty of Engineering, Helwan University, Cairo, 11795 Egypt; 3 Electrical engineering department, Faculty of Engineering, Badr University in Cairo (BUC), Cairo, 11829 Egypt

**Keywords:** Adaptive fuzzy logic controller, Fuzzy logic controllers, Proportional-Integral, Maximum power point tracking, Grid, And output power, Electrical and electronic engineering, Solar cells

## Abstract

This research validates An Adaptive Fuzzy Logic Controller (AFLC) has been developed for grid-connected photovoltaic (PV) systems. The primary objective of this implementation is to enhance the PV system’s power generation efficiency. For achieving this, techniques of Maximum Power Point Tracking (MPPT) are utilized, which are essential to extract the highest possible power outing from PV panels. Recent developments in MPPT methods focus on improving control strategies to ensure efficient operation and smooth integration with the grid. The performance of the AFLC is extensively evaluated and compared with other controllers, like fuzzy-logic controller (FLC) and Proportional Integral (PI). The proposed AFLC controller’s performance is evaluated with other methods to verify its effectiveness. To validate this method, the system is tested using MATLAB/Simulink simulations, along with experimental evaluations conducted on the control strategies are executed in real-time utilizing the DSpace DS1104 control. Experimental results show that the AFLC outperforms both the FLC and PI controllers in several key performance areas. Specifically, the AFLC demonstrates faster response times, higher convergence rates, decreased peak overshoot, minimal undershoot, and lower the error of the mean square. Additionally, the Compared to conventional Fuzzy Logic Control (FLC) and PI controllers, the AFLC delivers superior efficiency and transient response, and oscillation reduction. Compared to the FLC, the AFLC enhances tracking of power by 68.26%, and it achieves 86.25% improvement over the PI controller. These findings highlight the AFLC’s potential as a highly effective and reliable optimization tool for maximizing the output power of the systems of PV. Furthermore, integral absolute error (IAE) is used as a performance metric for the PV system connected to grid to assess the efficiency of the AFLC. The AFLC demonstrated superior performance over other methods, achieving a 20% increase in PV output power compared to traditional FLC and a 30% improvement over PI controllers. The errors of the PI, FLC and AFLC approaches, each utilizing five controllers, are estimated. The error of mean square is reduced by 79.67% in comparison to PI and by 66.5% in comparison to FLC.

## Introduction

Across the globe, renewable energy systems have gained significant popularity as a sustainable and eco-friendly replacement for traditional energy sources that rely on fossil fuels^1^. Because the sun is an abundant and long-lasting source of energy, power from solar is regarded as one of the most vital and sustainable renewable resources, not only for current energy needs but also for future generations^2^. Solar PV systems offer various advantages, inclusive straightforward installation, silent operation, minimal requirements of maintenance, unlimited availability, and environmental benefits. However, despite these advantages, PV systems face a major drawback related to the high initial costs required for purchasing and installing solar panels^3,4^. Additionally, since solar panels are unable to absorb all the sunlight that reaches them, their efficiency is typically limited to a range of approximately 15–18%^5,6^. The energy production of a PV system is significantly affected by solar irradiance and ambient temperature, both of which fluctuate with weather conditions, seasonal changes, and environmental influences^7,8^. MPPT and inverter control algorithms must be optimized to extract maximum current and ensure smooth grid integration under changing conditions. The MPPT algorithm tackles nonlinearities and fluctuations, maintaining stable and efficient PV system performance despite input variability^9,10^. One of the key difficulties in MPPT systems lies in accurately tracking the voltage and adjusting the duty cycle efficiently to harness the maximal power from the PV system^11–13^.

The essential role of MPPT controllers is for enhancing the output power of the modules of PV while accounting for *External conditions like changes in temperature and levels of solar exposure* levels. By optimizing power extraction, MPPT controllers contribute to higher energy yield and improved cost efficiency per watt^14,15^. Various MPPT control techniques have been studied and documented in the literatures, demonstrating their effectiveness in real-time operation. Most MPPT methods determine the optimal power output by dynamically adjusting the duty-cycle of the converter based on fluctuations in the array of the PV measured voltage and current. While all MPPT techniques share the common goal of maximizing energy efficiency, they differ in complexity, control parameters, implementation cost, stability at the maximum power point (MPP), application suitability, and tracking speed^9,13,17^.

Numerous conventional and advanced MPPT methods have been documented in the literature, primarily focusing on regulating the boost converter’s duty ratio and the resulting output voltage^18–20^. Among these, Techniques based on a fraction of the open-circuit voltage (FOCV) and short-circuit current (FSC) stand out due to their simplicity and ease of implementation^21^. However, these methods often struggle with inaccuracies in tracking the MPP and are generally limited to low-power applications. For scenarios requiring higher precision in MPP tracking, Additional traditional methods include Perturb and Observe (P&O)^22^, Incremental Conductance (IC)^23^, Hill Climbing (HC)^24^, state flow^25^, Kalman filter^26^, adaptive incremental conductance^27,28^, feedback control using power/current or power/voltage^29^, slider controller^30^, and incremental resistance^31^ are more suitable. These methods are applicable across both low- and high-power systems, offering improved accuracy in MPP tracking.

Li et al.^16^ developed an MPPT controller based on fuzzy logic to optimize power extraction from a PV system. The beta method was employed to enhance scheduling efficiency, especially during abrupt changes in temperature and irradiance. Zinelaabidine et al.^32^ performed a detailed comparison of multiple MPPT methods for PV systems, including P&O, INC, and FLC. MATLAB-Simulink simulations revealed that the INC method offered high precision, whereas the AC algorithm excelled in stability and minimized fluctuations, making it the top-performing technique. Metry et al.^33^ proposed a predictive control approach for MPPT in PV systems, removing the need for iterative current sensors usually associated with conventional algorithms. In^34^, researchers introduced a hybrid control strategy merging P&O with DC voltage regulation for grid-tied PV systems. The method effectively lowered harmonic distortion under 95 s and achieved near-unity power factor, as confirmed by real-time simulations. Authors in^35^, a hybrid MPPT algorithm was created by integrating P&O with open-circuit voltage and short-circuit current methods. This innovation enhanced tracking accuracy and system efficiency, achieving 99.5% steady-state efficiency—a 2% improvement over traditional P&O under diverse loads. Md. Naiem-Ur-Rahman and colleagues in^36^ present an innovative approach to maximum power point tracking (MPPT) in photovoltaic (PV) systems. The proposed controller integrates fuzzy logic with particle swarm optimization (PSO) to self-tune the MPPT process. It utilizes PV array voltage and power as inputs to generate the duty cycle for boost converters, eliminating the need for intermediate controllers like PID. Authors in^37^ present an experimental assessment of a type-2 fuzzy logic controller (T2FLC) for maximum power point tracking (MPPT) in solar energy systems under real environmental conditions. The controller demonstrates superior tracking efficiency and robustness compared to traditional MPPT methods, particularly in handling irradiance and temperature fluctuations, enhancing photovoltaic (PV) system performance. The adaptive type-2 fuzzy neural network controller designed to improve the dynamic efficiency of PV systems, evaluated using the EN 50,530 standard test^38^. The controller adapts to changing environmental conditions, outperforming classical methods by providing faster and more accurate MPPT responses, leading to better energy harvest in dynamic scenarios^39^.

Authors in^40^ develop a fuzzy-PI (proportional-integral) controller for MPPT in PV systems. The fuzzy-PI approach enhances tracking precision and stability during environmental changes, achieving improved power extraction compared to traditional PI controllers, especially under rapidly changing irradiance conditions.

This study proposes an enhancement for grid-connected PV systems by integrating AFLC into the control framework to maximize the power produced. The performance of AFLC is evaluated by MATLAB/SIMULINK 2024a in comparison to FLC and PI controller. The MPPT hardware configuration utilizes DSPACE for real-time control, with the control mechanism structured accordingly. This system is managed through the setup and incorporates the DSpace 1104 platform, which includes a digital signal processing (DSP) card integrated into a PC. Experimental validation was conducted in order to validate the efficiency of the proposed approach. However, The Adaptive Fuzzy Logic Controller (AFLC) which improves PV system connected to grid faces limitations. It requires precise modeling and tuning, which is challenging under dynamic conditions like rapid irradiance changes. Its computational complexity, particularly with many fuzzy rules, increases processing demands and hardware costs, restricting its use in low-resource systems. The study’s reliance on simulations and small-scale experiments may not fully reflect real-world, large-scale PV challenges. While the AFLC outperforms traditional FLCs and PI controllers, its performance compared to advanced methods like deep learning-based MPPT remains unexplored. These limitations highlight the need for future research to enhance scalability, computational efficiency, and real-world applicability for broader PV system integration.

The research introduces a robust and practical control strategy centered on AFLC technique to enhance the output power of the PV system connected to grid. Also, The AFLC system was also compared with the FLC and PI systems to prove the effectiveness of the system. and to demonstrate rapid convergence and improved performance in simulations. The key contributions of the study are outlined as follows:

1. AFLC performance is experimentally validated in real atmospheric conditions using the DS1104 platform, demonstrating its superiority over FLC and PI controllers.
2. The proposed AFLC technique is examined under various irradiance scenarios to establish its practical advantage over conventional FLC and PI methods.
3. Evaluation of the system using Matlab/Simulink 2024a, demonstrating the enhanced performance and robustness of AFLCs compared to FLC, and PI.
4. To show the viability of the AFLC methodology against FLC, and PIs techniques, the integral absolute error metric is employed to compare AFLC’s effectiveness with that of standard control methods.

The structure of this study is organized as follows: Section I presents an introduction to the topic and surveys related work. Section II explains the system setup in detail. Section III discusses simulation outcomes and their analysis. Section IV elaborates on the AFLC control board’s design and operation. Section V highlights experimental results derived using the DSpace 1104 platform. Lastly, Section VI offers concluding remarks and key findings.

### Model setup

**Fig. 1:**
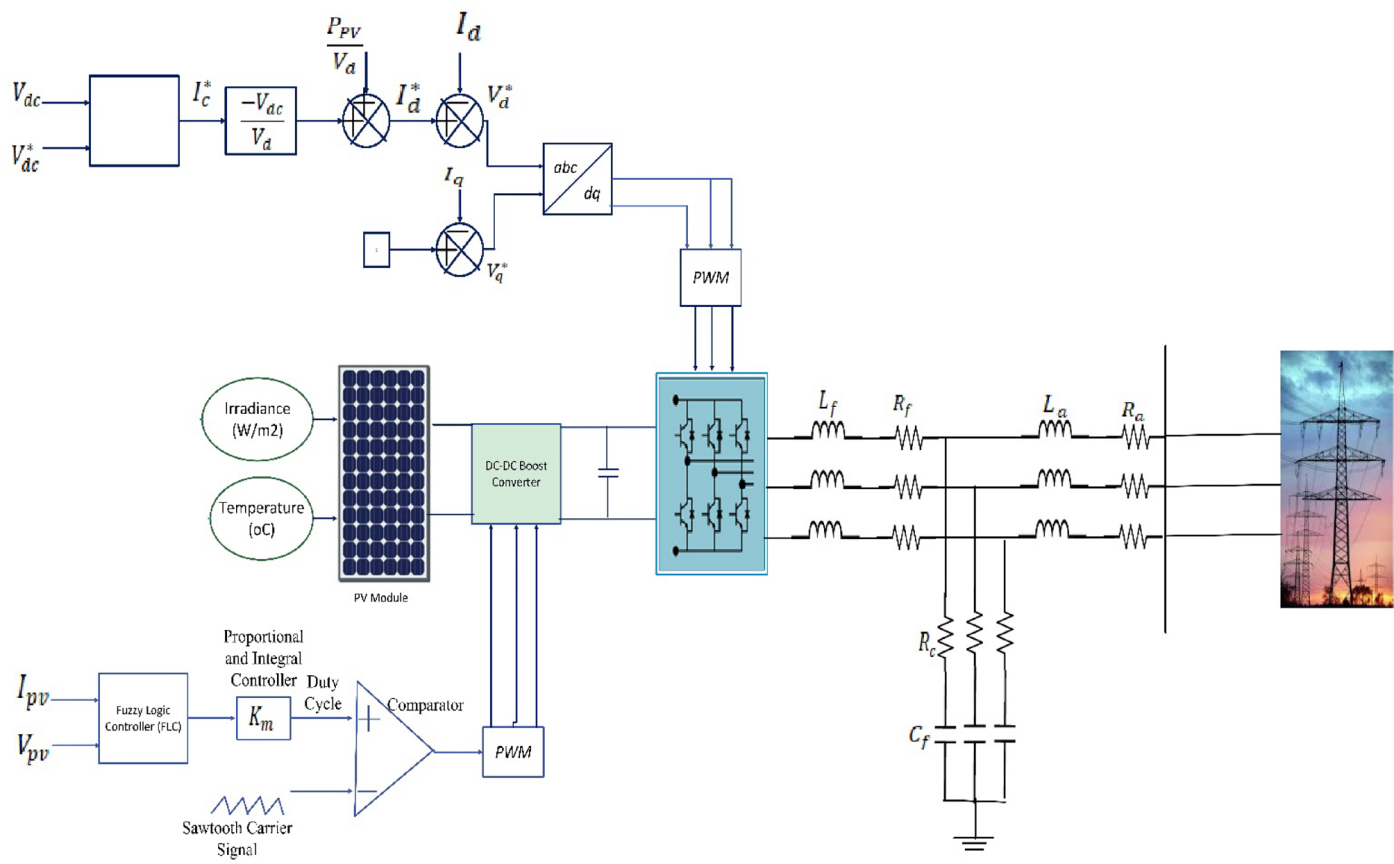
The schematic diagram of PV connected to grid system.

Figure 1 illustrates the grid-connected photovoltaic (PV) system model. The configuration includes a PV array, a DC/DC converter, a DC/AC inverter, an MPPT unit, filter components, and the grid interface.

## PV module modelling

A photovoltaic (PV) cell generates electrical energy from light through the photovoltaic effect. However, the output from a single PV cell is often insufficient to meet the power demands of electrical equipment. To overcome this limitation, multiple PV cells are connected in both series and parallel arrangements, which enhances the overall voltage and power output^41^. To accurately simulate the performance of a PV module, its mathematical model considers several parameters: the output voltage (V), output current (I), load resistance (R), the reverse saturation current (I_o_), the photocurrent generated by the module (I_ph_), the ideality factor (α), and the series resistance (R_s_). Critical specifications of a PV module include its maximum power output (P_max_), voltage at maximum power (V_mp_), current at maximum power (I_mp_), open-circuit voltage (V_oc_), and short-circuit current (I_sc_). When modeling a PV system in MATLAB, it’s essential to determine the current generated by solar irradiation—typically the short-circuit current (I_sc_) at a given ambient temperature (T_a_)^42–45^. The electrical behavior of a PV cell is primarily governed by five parameters: photocurrent (I_ph_), saturation current (I_o_), ideality factor (α), series resistance (R_s_), and shunt resistance (R_sh_)^46^:

1 $$\:{I}_{PV}={I}_{PV}-{I}_{d}-{I}_{sh}$$

Here, I_ph_ refers to the photocurrent (amperes), I_d_ is the current through the diode (in amperes), and I_sh_ denotes the current through the shunt resistance (also in amperes). These individual currents can be expressed mathematically as follows^47^:

2 $$\:\begin{array}{c}I=\:{I}_{ph}-{I}_{O}\left\{\text{exp}\left[\frac{e\left(V+I{R}_{s}\right)}{KTC}\right]-1\right\}-\frac{V+I{R}_{s}}{{R}_{sh}}\:\:\end{array}$$

3 $$\:\begin{array}{c}I=\:{I}_{ph}-{I}_{D}\:=\:{I}_{ph}-{I}_{O}\left[\text{exp}\left[\frac{eV}{KTC}\right]-1\right]\end{array}$$

4 $$\:\begin{array}{c}{P}_{max}={I}_{max}\times\:{V}_{max}\:\end{array}$$

5 $$\:\begin{array}{c}{P}_{max}={I}_{SC}\times\:{V}_{OC}\times\:FF\:\end{array}$$

6 $$\:\begin{array}{c}FF=\frac{{P}_{max}}{{I}_{SC}}\times\:{V}_{OC}={I}_{max}\times\:\frac{{V}_{max}}{{I}_{SC}}\times\:{V}_{OC}\end{array}$$

7 $$\:\begin{array}{c}\text{exp}\left[\frac{e{V}_{OC}}{KTC}\right]-1=\frac{{I}_{SC}}{{I}_{O}}\:\end{array}$$

The equation is solved for V_oc_:

8 $$\:\begin{array}{c}{V}_{oc}=\frac{KTC}{e}ln\left(\frac{{I}_{sc}}{{I}_{o}}+1\right)={V}_{t}ln\left(\frac{{I}_{sc}}{{I}_{o}}+1\right)\:\end{array}$$

where V_t_​, the thermal voltage (in volts), is defined as

9 $$\:\begin{array}{c}{V}_{t}=\frac{KTC}{e}\:\end{array}$$

The output power of the PV cell is obtained from:

10 $$\:\begin{array}{c}P=I*V\:\end{array}$$

Assuming V = I⋅R, the output power depends on the load resistance R, yielding Substituting Eq. (2) into Eq. (11) gives:

11 $$\:\begin{array}{c}P={I}^{2}*R\:\end{array}$$

By inserting Eq. (2) into Eq. (11), the resulting expression becomes:

12 $$\:\begin{array}{c}P=\left\{{I}_{sc}-{I}_{o}\left[\text{exp}\left(\frac{eV}{KTC}\right)-1\right]\right\}V\:\end{array}$$

To find the optimal condition, Eq. (12) can be differentiated with respect to VV, and the derivative is set to zero.

13 $$\:\begin{array}{c}exp\left(\frac{{eV}_{max}}{KTC}\right)\left(1+\frac{{eV}_{max}}{KTC}\right)=1+\frac{{I}_{sc}}{{I}_{o}}\end{array}$$

14 $$\:\begin{array}{c}{I}_{max}={I}_{sc}-{I}_{0}\left[{e}^{\left(\frac{ev}{KTC}\right)}-1\right]={I}_{sc}-{I}_{0}\left[\frac{1+\frac{{I}_{sc}}{{I}_{o}}}{1+\frac{e{V}_{max}}{KTC}}-1\right]\end{array}$$

that also outcomes:

15 $$\:\begin{array}{c}{I}_{max}=\frac{{eV}_{max}}{KTC+{eV}_{max}}\left({I}_{sc}+{I}_{o}\right)\end{array}$$

By using Eq. (10),

16 $$\:\begin{array}{c}{P}_{max}\:=\frac{\text{e}{V}_{max}^{2}}{KTC+{eV}_{max}\:}\left({I}_{sc}+{I}_{o}\right)\:\end{array}$$

17 $$\:\begin{array}{c}\:{\eta\:}_{max}\:=\frac{{P}_{max}}{{P}_{in}}=\frac{{I}_{max}{V}_{max}}{{AG}_{t}}\:\end{array}$$

## Power control techniques

### Active reactive control

Active power is delivered through current vectors aligned with the voltage vector (u), while reactive power is generated by the current vectors associated with $\:{V}_{\perp\:}.\:{V}_{\perp\:}\:$is thermal voltage, this concept can be expressed as:

18 $$\:{i}_{p}=\frac{P}{|V{|}^{2}}V,\:\:{i}_{q}=\frac{Q}{|V{|}^{2}}{V}_{\perp\:}$$

where $\:|V{|}^{2}$ represents the magnitude of the grid voltage vector and is defined by the following:

19 $$\:|V{|}^{2}={\left|{V}^{+}\right|}^{2}+{\left|{V}^{-}\right|}^{2}+2\left|{V}^{+}\right|\left|{V}^{-}\right|cos\left(2\omega\:t+{\phi\:}^{+}-{\phi\:}^{-}\right)$$

The reference current can be expressed as:

20 $$\:i={i}_{p}+{i}_{q}=\frac{PV+Q{V}_{\perp\:}}{{\left|{V}^{+}\right|}^{2}+2{V}^{+}*{V}^{-}+{\left|{V}^{-}\right|}^{2}}\:\:$$

As a result, the inverter output power using the Active Reactive Control (ARC) method can be calculated as:

21 $$\:\begin{array}{c}p=V*i=V\left(\frac{P}{|V{|}^{2}}*V+\frac{Q}{|V{|}^{2}}*{V}_{\perp\:}\right)=P\end{array}$$

22 $$\:\:q={V}_{\perp\:}*i={V}_{\perp\:}\left(\frac{P}{|\:V{|}^{2}}*V+\frac{Q}{|\:V{|}^{2}}*{\:V}_{\perp\:}\right)=Q\:\:$$

Although the IARC technique enables precise generation of the power reference, the current reference is not purely sinusoidal due to ripples of 120 Hz present in ∣V∣^2^.

23 $$\:\begin{array}{c}{i}_{p}^{*}\:=g*v\end{array}$$

24 $$\:\begin{array}{c}{i}_{q}^{*}\:=b*{v}_{\perp\:}\end{array}$$

25 $$\:\:{i}_{p}^{*}*v\:=gv\cdot\:v=P\Rightarrow g|v{|}^{2}=Pg=\frac{P}{|v{|}^{2}}$$

26 $$\:{i}_{q}^{*}*{v}_{\perp\:}\:=b{v}_{\perp\:}\cdot\:{v}_{\perp\:}=Q\Rightarrow b|v{|}^{2}\Rightarrow b=\frac{Q}{|v{|}^{2}}$$

27 $$\:\begin{array}{c}\:{i}_{p}^{*}\:=\frac{P}{|v{|}^{2}}v\end{array}$$

28 $$\:\begin{array}{c}\:{i}_{q}^{*}\:=\frac{Q}{|v{|}^{2}}{v}_{\perp\:}\end{array}$$

29 $$\:\begin{array}{c}{i}^{*}\:={i}_{p}^{*}+{i}_{q}^{*}\end{array}$$

30 $$\:|v{|}^{2}\:={v}_{a}^{2}+{v}_{b}^{2}+{v}_{c}^{2}={v}_{\alpha\:}^{2}+{v}_{\beta\:}^{2}={v}_{d}^{2}+{v}_{q}^{2}={m}^{2}\:$$

### Average active-reactive control

The Average Active Reactive Control (AARC) method employs $\:{V}_{\varSigma\:}^{2}$ instead of $\:|V{|}^{2}$ to calculate the current reference, effectively reducing harmonics that are present in the current reference derived from the ARC approach. The value of $\:{V}_{\varSigma\:}^{2}$​ is determined as follows:

31 $$\:\begin{array}{c}{V}_{\varSigma\:}^{2}={\left|{V}^{+}\right|}^{2}+{\left|{V}^{-}\right|}^{2}\end{array}$$

The current reference represented as

32 $$\:\begin{array}{c}i={i}_{p}+{i}_{q}=\frac{PV+{QV}_{\perp\:}}{{\left|{V}^{+}\right|}^{2}+{\left|{V}^{-}\right|}^{2}}\end{array}$$

Additionally, the inverter power output is:

33 $$\:\begin{array}{c}p=P\left[1+\frac{2\left|{V}^{+}\right|\left|{V}^{-}\right|}{{\left|{V}^{+}\right|}^{2}+{\left|{V}^{-}\right|}^{2}}\text{cos}\left(2\omega\:t+{\varphi\:}^{+}-{\varphi\:}^{-}\right)\right]\end{array}$$

34 $$\:\begin{array}{c}\:q=Q\left[1+\frac{2\left|{V}^{+}\right|\left|{V}^{-}\right|}{{\left|{V}^{+}\right|}^{2}+{\left|{V}^{-}\right|}^{2}}\text{cos}\left(2\omega\:t+{\varphi\:}^{+}-{\varphi\:}^{-}\right)\right]\end{array}$$

As indicated by (33) and (34), p and q is active and reactive power, the current reference is typically sinusoidal; however, the power output exhibits fluctuations at a frequency of 120 Hz.

35 $$\:\begin{array}{c}{i}_{p}^{*}=Gv;\:\:\:\:\:\:\:\:\:\:\:\:\:\:\:\:\:G=\frac{P}{{V}_{\varSigma\:}^{2}}\:\end{array}$$

36 $$\:\begin{array}{c}{i}_{q}^{*}=Bv;\:\:\:\:\:\:\:\:\:\:\:\:\:\:\:\:\:\:\:\:B=\frac{Q}{{V}_{\varSigma\:}^{2}}\:\end{array}$$

37 $$\:\begin{array}{c}{V}_{\varSigma\:}\:=\sqrt{\frac{1}{T}}{\int\:}_{0}^{T}|U{|}^{2}dt\:\:=\sqrt{{\left|{U}^{+}\right|}^{2}}+{\left|{U}^{-}\right|}^{2}\end{array}$$

38 $$\:\begin{array}{c}\:p\:={i}_{p}^{*}\cdot\:u=\frac{|u{|}^{2}}{{U}_{\varSigma\:}^{2}}p=p+p{\check{}}\end{array}$$

39 $$\:\begin{array}{c}\:p\:=p\left[1+\frac{2\left|{v}^{+}\right|\left|{v}^{-}\right|}{{\left|{v}^{+}\right|}^{2}+{\left|{v}^{-}\right|}^{2}}\text{cos}\left(2\omega\:t+{\phi\:}^{+}-{\phi\:}^{-}\right)\right]\end{array}$$

40 $$\:\begin{array}{c}\:q\:={i}_{q}^{*}\cdot\:{v}_{\perp\:}=\frac{|v{|}^{2}}{{V}_{\varSigma\:}^{2}}Q=Q\end{array}$$

41 $$\:\begin{array}{c}\:q\:=Q\left[1+\frac{2\left|{v}^{+}\right|\left|{v}^{-}\right|}{{\left|{v}^{+}\right|}^{2}+{\left|{v}^{-}\right|}^{2}}\text{cos}\left(2\omega\:t+{\phi\:}^{+}-{\phi\:}^{-}\right)\right]\end{array}$$

### Reparations for positive sequence and negative sequence

The Positive and Negative Sequence Control (PNSC) method produces unbalanced currents by computing reference values that account for components for both positive sequence and negative sequence. It is observed that, during voltage sag events, maintaining only a single power set-point can help reduce power oscillations. To achieve this, specific constraints are applied:

42 $$\:\begin{array}{c}{P}^{*}={{U}^{-}.{i}_{p}^{-}+U}^{+}.{i}_{p}^{+}\Rightarrow\:0={{U}^{-}.{i}_{p}^{+}+U}^{+}.{i}_{p}^{-}\Rightarrow\:{i}_{q}=0\end{array}$$

43 $$\:\begin{array}{c}\:{Q}^{*}={{U}_{\perp\:}^{-}.{i}_{q}^{-}+U}_{\perp\:}^{+}.{i}_{q}^{+}\Rightarrow\:0={{U}_{\perp\:}^{-}.{i}_{q}^{+}+U}_{\perp\:}^{+}.{i}_{q}^{-}\Rightarrow\:{i}_{p}=0\end{array}$$

From Eqs. (42) and (43), where $\:{P}^{*}$, $\:{Q}^{*}$are desired active and reactive power, it is essential to eliminate interactions between voltage and current components of differing sequences, as these interactions affect the average instantaneous power-primarily based on the multiplication of voltages and currents within the same sequence. Consequently, the vectors for active and reactive currents are defined as follows:

44 $$\:\begin{array}{c}\:{i}_{p}^{*}=\frac{{P}^{*}}{{\left|{U}^{+}\right|}^{2}-{\left|{U}^{-}\right|}^{2}}\left({U}^{+}-{U}^{-}\right)={g}^{\mp\:}\left({U}^{+}-{U}^{-}\right)\:\end{array}$$

45 $$\:\begin{array}{c}\:{i}_{q}^{*}=\frac{{Q}^{*}}{{\left|{U}^{+}\right|}^{2}-{\left|{U}^{-}\right|}^{2}}\left({U}_{\perp\:}^{+}-{U}_{\perp\:}^{-}\right)={b}^{\mp\:}\left({U}_{\perp\:}^{+}-{U}_{\perp\:}^{-}\right)\end{array}$$

In this context, b±​ and g±​ represent the instantaneous positive/negative conductivity of PNSC and its validation model and verification, respectively. The first objective is to reduce the components of fluctuations in both active power and reactive power. However, the sine components remain, meaning power oscillations persist for any non-zero power set-points. Calculations (44) and (45) highlight a unique scenario where ∣U+∣=∣U−∣∣U+​∣=∣U−​∣, indicating the point which the voltages positive sequence and negative sequence are equal. This implies that under conditions of severe voltage imbalance, it is theoretically impossible to eliminate power fluctuations.

46 $$\:\begin{array}{c}{i}^{*}={i}^{*+}+{i}^{*-}\end{array}$$

47 $$\:\begin{array}{c}{U}^{-}.{i}_{p}^{*-}+{U}^{+}.{i}_{p}^{*+}=P\end{array}$$

48 $$\:\begin{array}{c}{U}^{-}.{i}_{p}^{*+}+{U}^{+}.{i}_{p}^{*-}=0\end{array}$$

49 $$\:\begin{array}{c}\:{U}^{+}.{i}_{p}^{*-}=-{U}^{-}.{i}_{p}^{*+}\Rightarrow{\left|{U}^{+}\right|}^{2}.{i}_{p}^{*-}=-{v}^{+}\cdot\:{i}_{p}^{*+}.{U}^{-}\end{array}$$

50 $$\:\begin{array}{c}\:\:\Rightarrow{i}_{p}^{*-}=-\frac{{U}^{+}.{i}_{p}^{*+}}{{\left|{U}^{+}\right|}^{2}}{U}^{-}\end{array}$$

$$\:\begin{array}{c}\:\:P={U}^{+}.{i}_{p}^{*+}\left(1-\frac{{\left|{U}^{-}\right|}^{2}}{{\left|{U}^{+}\right|}^{2}}\right)\end{array}$$

51 $$\:\begin{array}{c}\:\:P={\left|{U}^{+}\right|}^{2}\cdot\:{i}_{p}^{*+}\Rightarrow{i}_{p}^{*+}\end{array}$$

52 $$\:\begin{array}{c}\:\:=\frac{P}{{\left|{U}^{+}\right|}^{2}-{\left|{U}^{-}\right|}^{2}}{U}^{+}\end{array}$$

53 $$\:\begin{array}{c}\:{U}_{\perp\:}^{+}\cdot\:{i}_{q}^{*+}+{U}_{\perp\:}^{-}\cdot\:{i}_{q}^{*-}=Q\end{array}$$

54 $$\:\begin{array}{c}\:{U}_{\perp\:}^{+}\cdot\:{i}_{q}^{*-}+{U}_{\perp\:}^{-}\cdot\:{i}_{q}^{*+}=0\end{array}$$

55 $$\:\begin{array}{c}\:{i}^{*}={i}_{p}^{*}+{i}_{q}^{*}\end{array}$$

56 $$\:\begin{array}{c}\:\:={g}^{\pm\:}\left({U}^{+}-{U}^{-}\right)+{b}^{\pm\:}\left({U}_{\perp\:}^{+}-{U}_{\perp\:}^{-}\right)\end{array}$$

57 $$\:\begin{array}{c}\:{i}^{+}={i}_{p}^{+}+{i}_{q}^{+}\end{array}$$

58 $$\:\begin{array}{c}\:{i}^{-}={i}_{p}^{-}+{i}_{q}^{-}\end{array}$$

$$\:\begin{array}{c}\:P={\underbrace{{U}^{+}\cdot\:{i}_{p}^{+}+{U}^{-}\cdot\:{i}_{p}^{-}}}_{p}+\:{\underbrace{{U}^{+}\cdot\:{i}_{q}^{+}+{U}^{-}\cdot\:{i}_{q}^{-}}}_{0}\end{array}$$

$$\:\begin{array}{c}\:\:+{\underbrace{{U}^{+}\cdot\:{i}_{p}^{-}+{U}^{-}\cdot\:{i}_{p}^{+}}}_{0}\end{array}$$

59 $$\:\begin{array}{c}\:\:+{\underbrace{{U}^{+}\cdot\:{i}_{q}^{-}+{U}^{-}\cdot\:{i}_{q}^{+}}}_{\stackrel{-}{p}}\end{array}$$

$$\:\begin{array}{c}\:Q={\underbrace{{U}_{\perp\:}^{+}\cdot\:{i}_{q}^{+}+{U}_{\perp\:}^{-}\cdot\:{i}_{q}^{-}}}_{Q}+{\underbrace{{U}_{\perp\:}^{+}\cdot\:{i}_{p}^{+}+{U}_{\perp\:}^{-}\cdot\:{i}_{p}^{-}}}_{0}\end{array}$$

$$\:\begin{array}{c}\:+{\underbrace{{U}_{\perp\:}^{+}\cdot\:{i}_{q}^{-}+{U}_{\perp\:}^{-}\cdot\:{i}_{q}^{+}}}_{0}\end{array}$$

60 $$\:\begin{array}{c}\:\:+{\underbrace{{U}_{\perp\:}^{+}\cdot\:{i}_{p}^{-}+{U}_{\perp\:}^{-}\cdot\:{i}_{p}^{+}}}_{\stackrel{-}{p}}\end{array}$$

### Balanced positive sequence control

The control of balanced positive sequence generates a set of fully balanced positive currents sequence sinusoidal. These reference currents are calculated using solely the positive sequence voltage vector.

61 $$\:\begin{array}{c}{i}_{p}^{*}=\frac{{P}^{*}}{{\left|{U}^{+}\right|}^{2}}{U}^{+}={G}^{+}{U}^{+}\end{array}$$

62 $$\:\begin{array}{c}{i}_{q}^{*}=\frac{{Q}^{*}}{{\left|{U}^{+}\right|}^{2}}{U}_{\perp\:}^{+}={B}^{+}{U}_{\perp\:}^{+}\end{array}$$

63 $$\:\begin{array}{c}P=u.{i}_{p}^{*}={\underbrace{{u}^{+}\cdot\:{i}_{p}^{*}}}_{p}+{\underbrace{{u}^{-}\cdot\:{i}_{p}^{*}}}_{\stackrel{-}{p}}\end{array}$$

64 $$\:\begin{array}{c}\:q={u}_{\perp\:}.{i}_{q}^{*}={\underbrace{{u}_{\perp\:}^{+}\cdot\:{i}_{q}^{*}}}_{Q}+{\underbrace{{u}_{\perp\:}^{+}\cdot\:{i}_{q}^{*}}}_{\stackrel{-}{q}}\end{array}$$

The current outputs are generally aligned with the positive sequence of the voltage and are influenced by the positive sequence conductance (G⁺) as well as a parameter derived from computational neuroscience (B⁺) remaining constant. As a result, the Balanced Positive Sequence Control (BPSC) generates sinusoidal currents via the positive sequence. However, this approach fails to eliminate power fluctuations except in cases where P* and Q* are both zero.

**Fig. 2:**
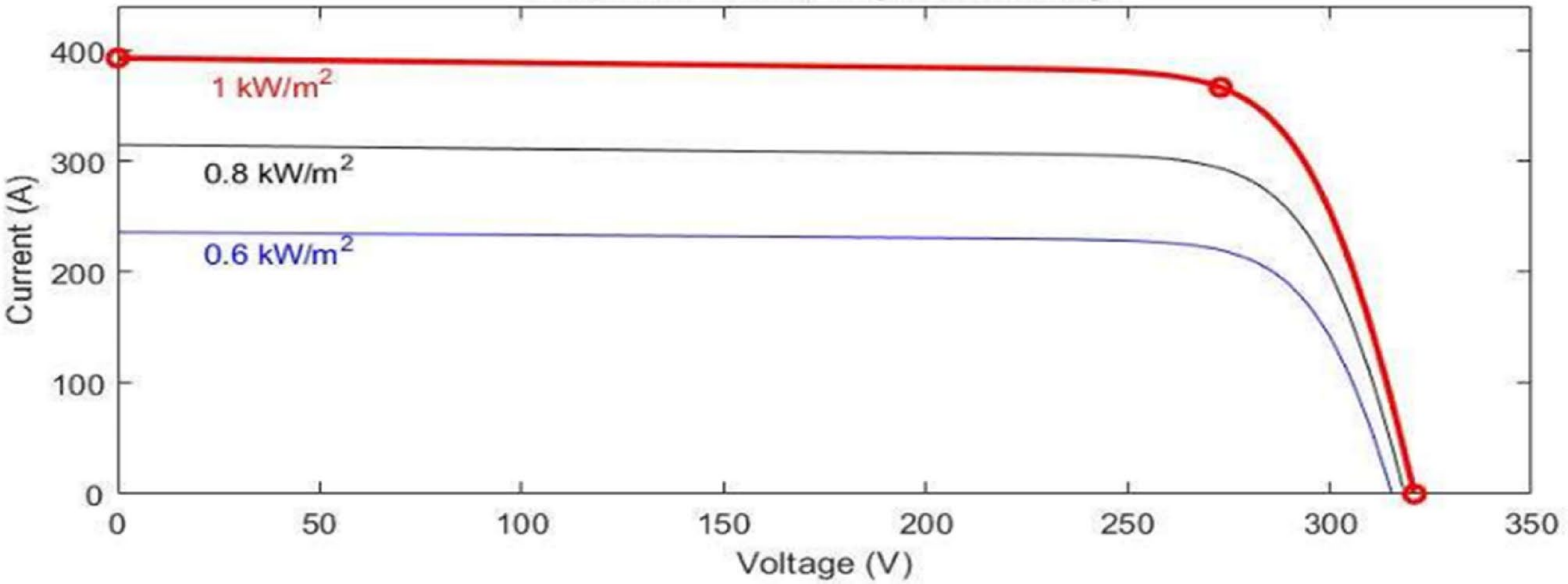
P-V curves under changing solar radiation.

**Fig. 3:**
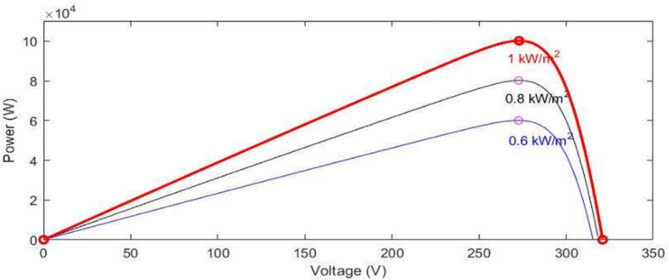
P-V curves under changing temperature.

The PV array module incorporates two inputs to account for changing conditions of weather. Input 1 represents solar irradiance in W/m², and input 2 corresponds to temperature in degrees Celsius. The PV module’s performance is modeled and analyzed using MATLAB. In Fig. 2, the current-voltage characteristics of the module are simulated for varying levels of radiation from solar, ranging from 700 to 1100 W/m², but the temperature is maintained at a constant 25 °C. Conversely, Fig. 3 illustrates the P-V curves for the same module at different radiation, varying from 200 to 1000 W/m², with the temperature fixed at 25 °C.

This study utilizes the SunPower SPR-305 PV module. The system discussed comprises an array of photovoltaic. Specifically, the PV array with 100-kW is composed of 66 strings and five modules connected in series (resulting in 66 × 5 × 305 W = 100 kW). Table 1 outlines the manufacturer specifications for the module The module’s electrical characteristics are recorded under standard test conditions (STC), specifically 1000 W/m² of solar irradiance and an ambient temperature of 25 °C.

**Table 1:** PV system Specifications.

Parameter	Value
**PV model**	SunPower SPR-305
**Number of Cells**	5 modules in series, 66 string in parallel
**Maximum Power**	305 W/One Module
**Voltage Open Circuit**	64.2 V
**Current Short Circuit**	5.96 A
**Voltage Max. Power**	54.6 V
**Current Max. Power**	5.56 A

### DC boost converter

This converter is a system that elevates the output voltage to a higher level by amplifying the correct voltage at the converter’s input^48^. Its integration into power electronics conversion circuits is crucial to optimize the energy generated and efficiently utilize photovoltaic systems. Importantly, the application of DC-DC converters significantly enhances the performance of power generated by renewable energy sources^49^. Since individual photovoltaic cells typically generate lower voltage levels than what is required by the inverter’s input stage, a DC-DC boost converter is employed to elevate the voltage accordingly.

### Inverter

A Voltage Source Inverter (VSI) plays a vital role in converting constant DC voltage into three-phase AC voltage^50^. It enables the transformation of maximum extracted power from the DC-DC converter into usable AC output. In this work, a three-level, three-phase VSI is carefully designed to channel power from the PV system into the electrical grid^51^. It converts a 500 V DC supply into a 220 V AC output while maintaining a unity power factor.

### Fuzzy logic controller

**Fig. 4:**
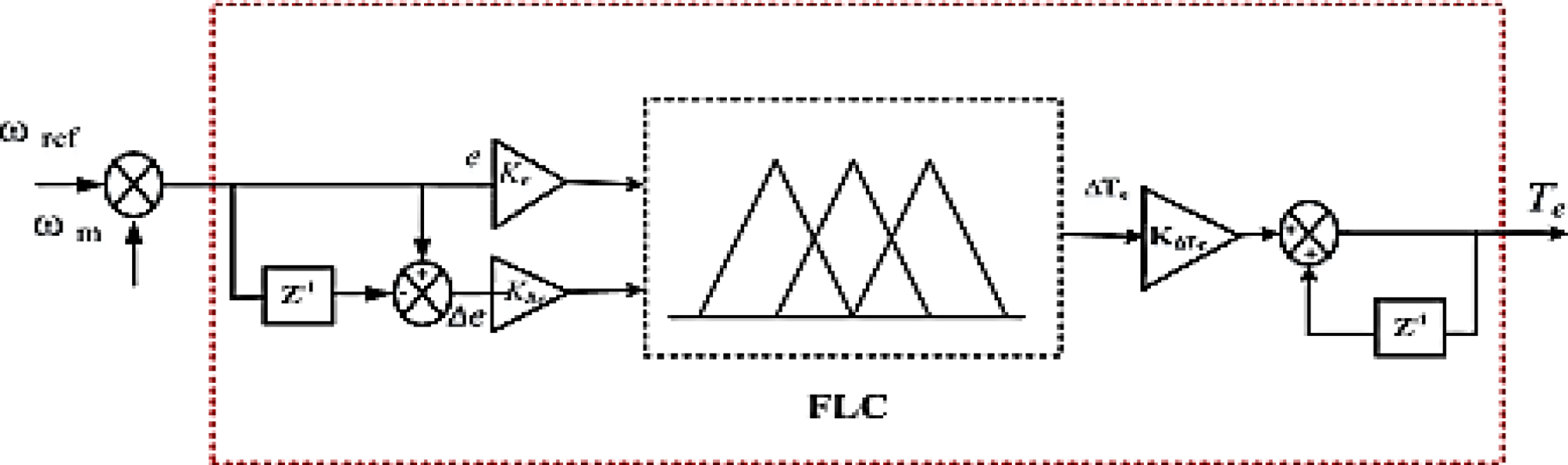
FLC Structure.

Fuzzy Logic Control (FLC) is a control approach that utilizes fuzzy reasoning to manage system behavior and derive decisions under uncertainty^52,53^. The configuration of the FLC is illustrated in Fig. 4.

### Adaptive fuzzy logic controller

**Fig. 5:**
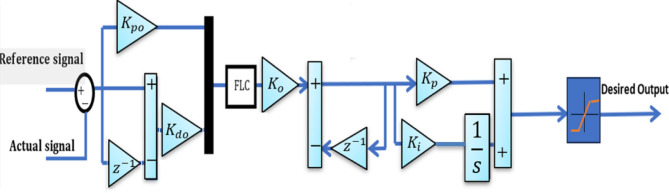
AFLC Structure.

**Fig. 6:**
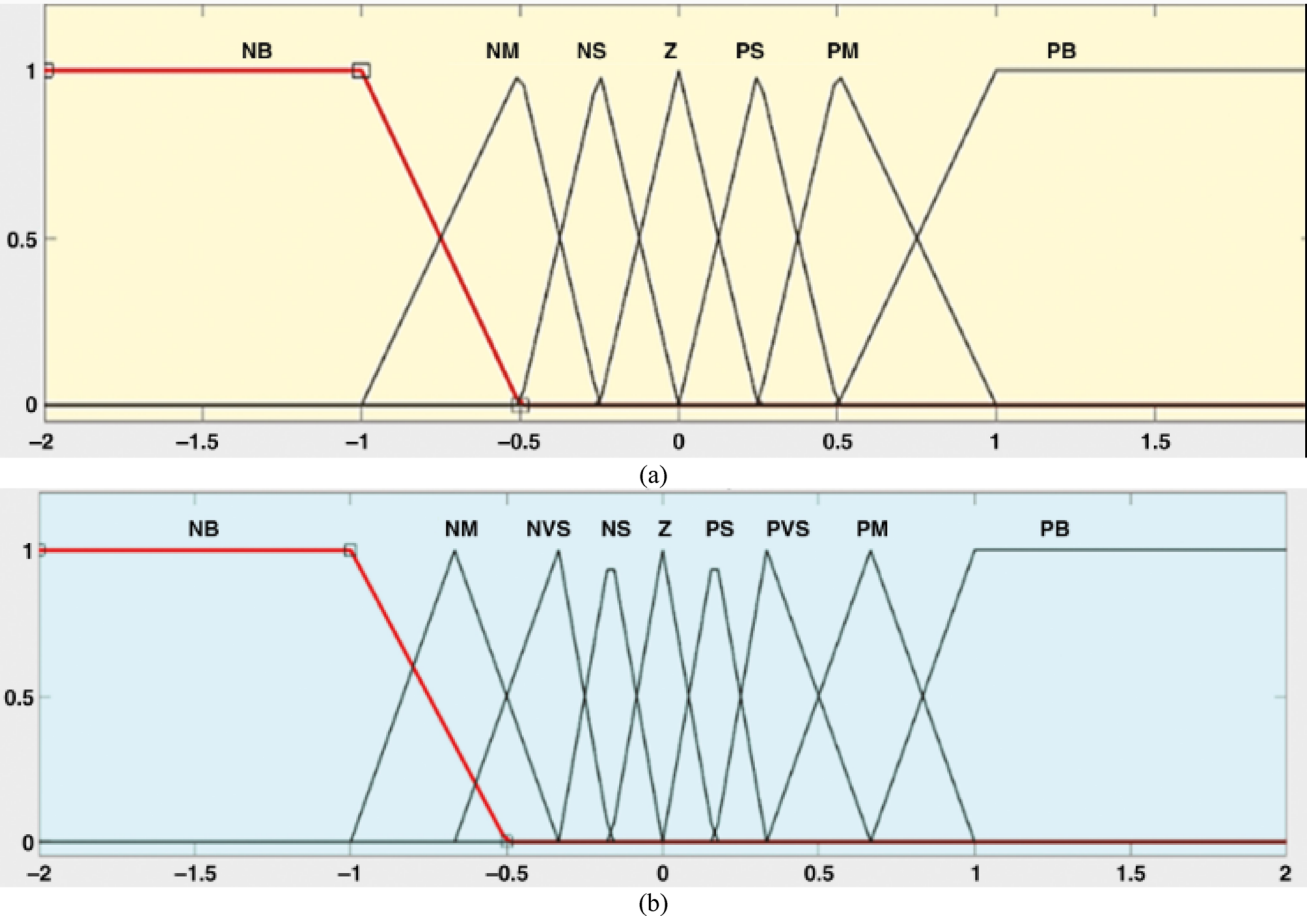
Membership Functions; (**a**) for inputs; and (**b**) output.

 Conventional fuzzy logic controllers are often constrained by limited input and output ranges. To overcome this issue, a specialized control mechanism is implemented to modify the gain applied to fuzzy logic inputs. This mechanism is known as the Adaptive Fuzzy Logic Controller (AFLC), and it consists of fuzzification, a rule-based fuzzy logic core, and a defuzzification stage^54^. The AFLC integrated with a PI controller is illustrated in Fig. 5. According to Fig. 6, the system’s linguistic terms are defined as follows: Positive Big (PB), Big (BB), Negative Big (NB), Negative Medium (NM), Negative Small (NS), and Zero (Z). A commonly referenced method proposes the use of triangular membership functions (MFs) that feature overlapping zones (PB) to define fuzzy input and output sets^54–56^. The fuzzy reasoning system applies 49 predefined control rules to generate an output that achieves a balance between computational efficiency and accuracy, as detailed in Table 2^57,58^. These control rules are formulated by analyzing the trade-off between the prediction precision and the computational load associated with the AFLC framework. In cases where no limitations are imposed, increasing the number of fuzzy sets associated with each input variable leads to more rules being simultaneously activated. This effect arises because each input is broken into a greater number of fuzzy categories, which is directly related to how much the fuzzy sets overlap with each other^57^. The design ensures that the controller remains accurate while not overwhelming computational resources, making AFLC an advanced and efficient alternative to traditional fuzzy logic systems when dealing with dynamic or uncertain control scenarios.

**Table 2:** AFLC rule base.

e	NS	NM	NB	PB	PS	PM	Z
Δe							
**NB**	NB	NB	NB	Z	NS	NVS	NM
**PS**	Z	NVS	NS	PB	PS	PM	PVS
**NS**	NS	NM	NB	PS	Z	PVS	NVS
**NM**	NM	NB	NB	PVS	NVS	Z	NS
**Z**	NVS	NS	NM	PM	PVS	PS	Z
**PB**	PS	PVS	Z	PB	PB	PB	PM
**PM**	PVS	Z	NVS	PB	PM	PB	PS

## Simulation results and discussion

In this study, a MATLAB/Simulink 2024a model of a grid-tied PV system was developed. A step change in solar irradiance was applied to evaluate the performance and robustness of the AFLC in comparison to traditional FLC and PI controllers. Table 1 provides the specifications of the system. Simulation outcomes under various conditions demonstrate the superior performance of the AFLC. Results show clear performance enhancements when compared with conventional controllers. The performance of all three controllers is evaluated through simulation analysis as described below.

**Case I: Step Solar Irradiance**

**Fig. 7:**
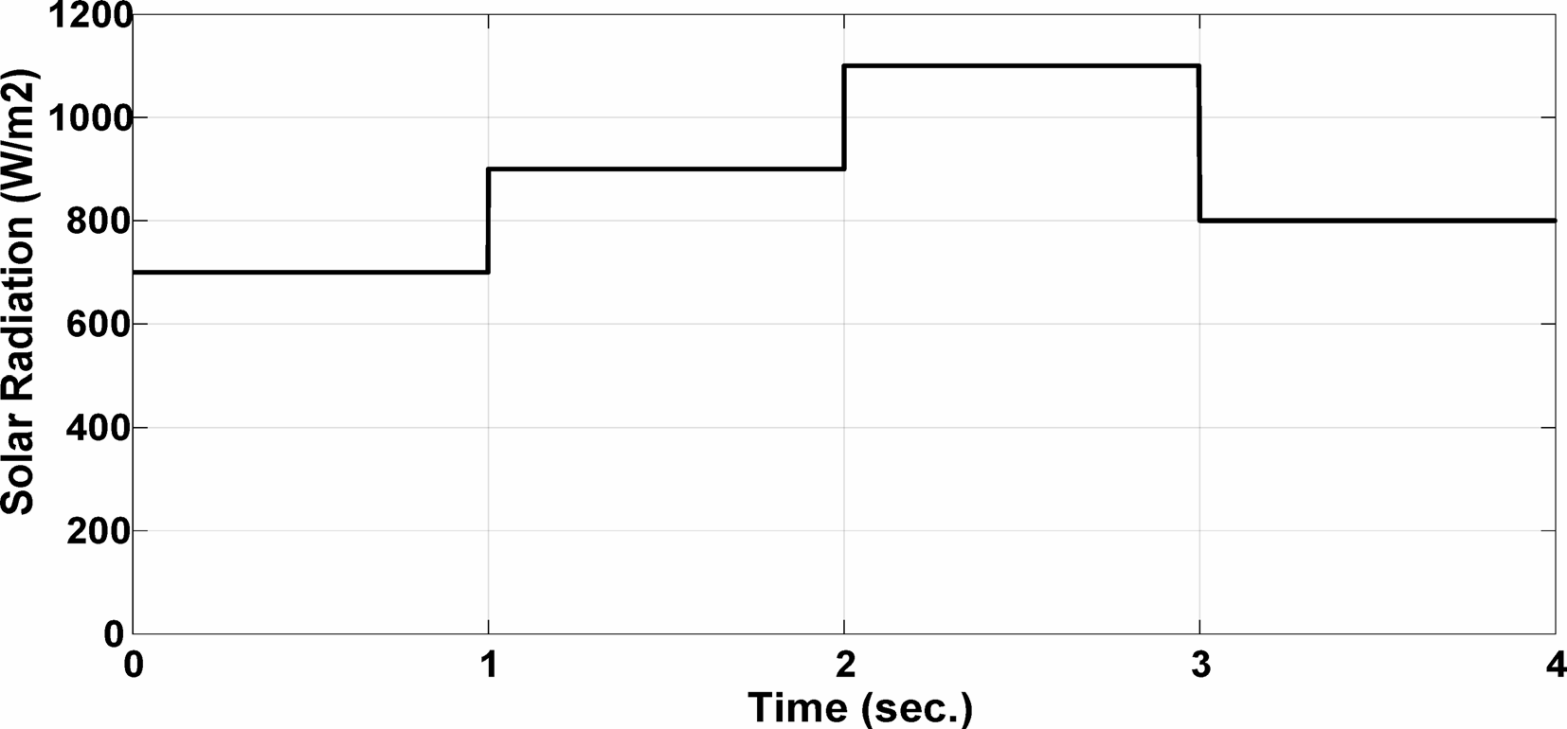
Solar Radiation.

**Fig. 8:**
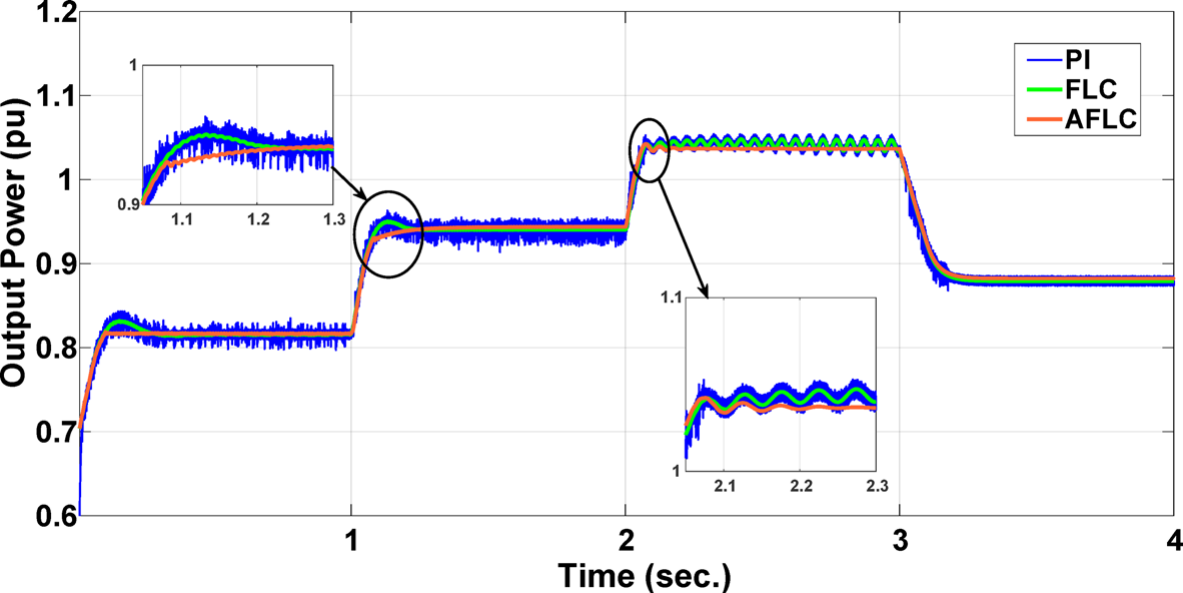
Output Power.

**Fig. 9:**
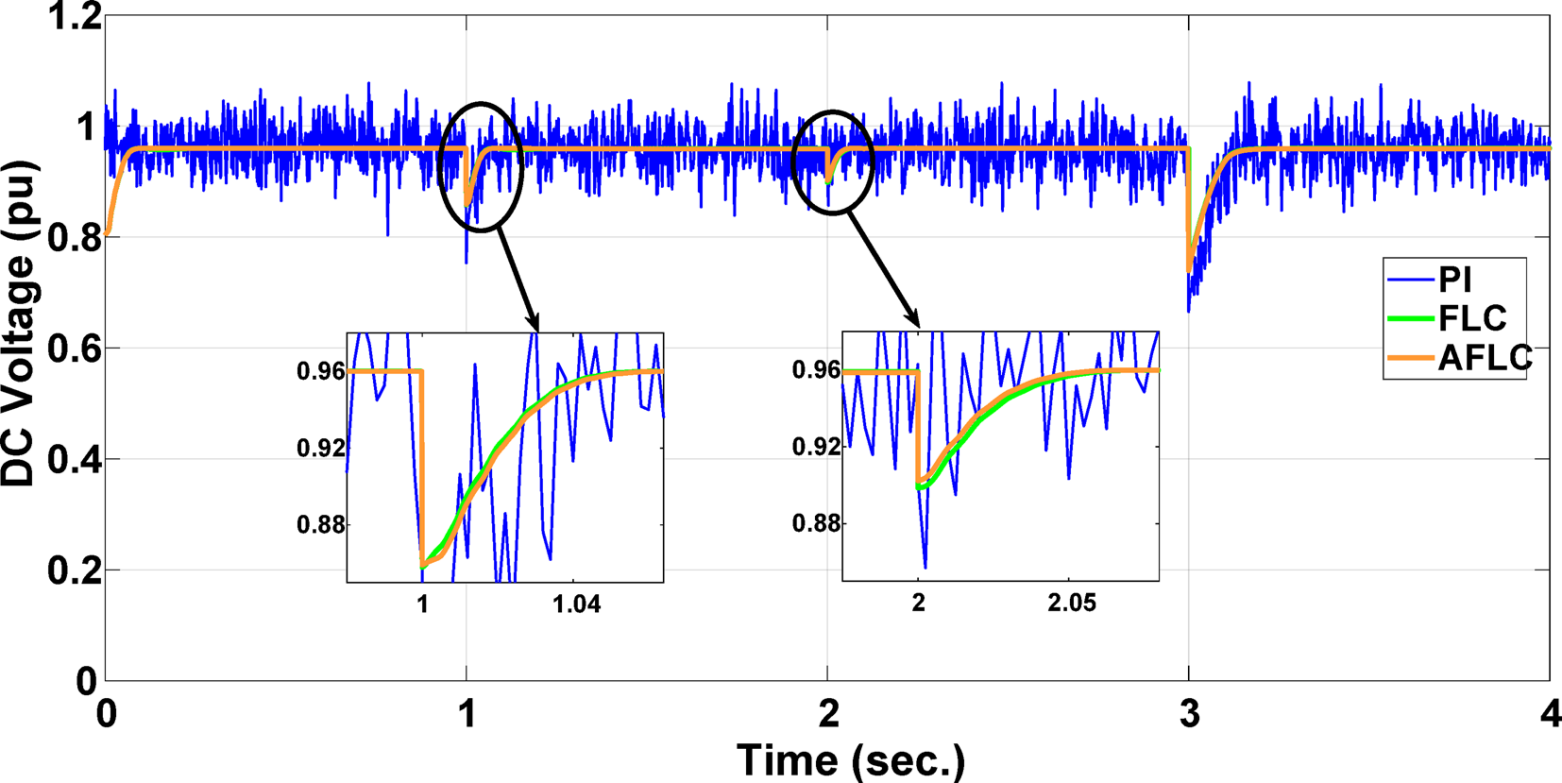
DC Voltage.

**Fig. 10:**
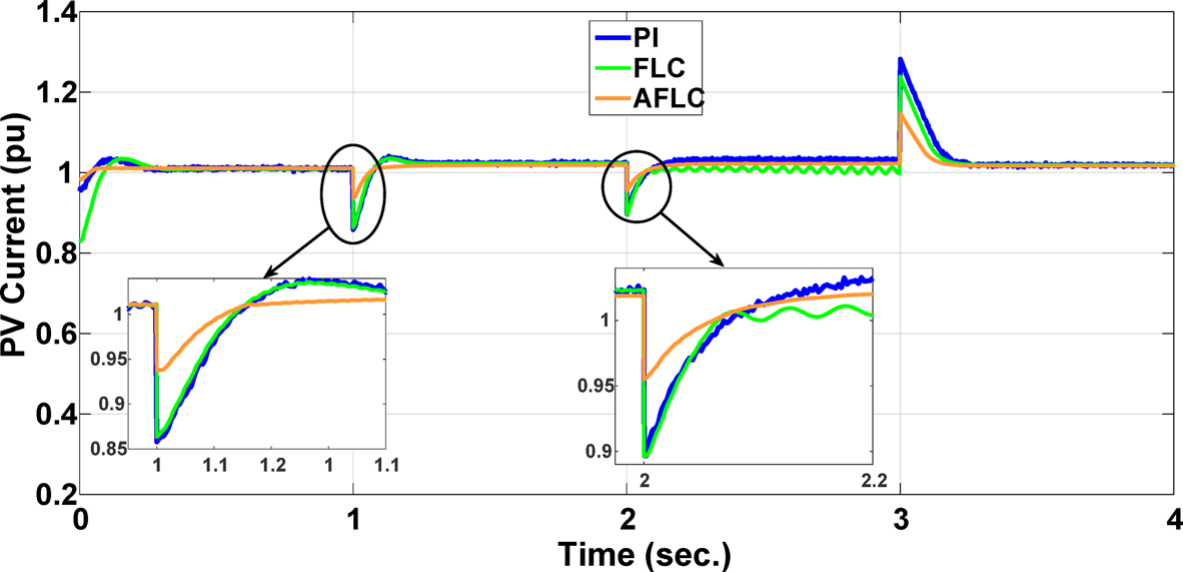
PV Current.

**Fig. 11:**
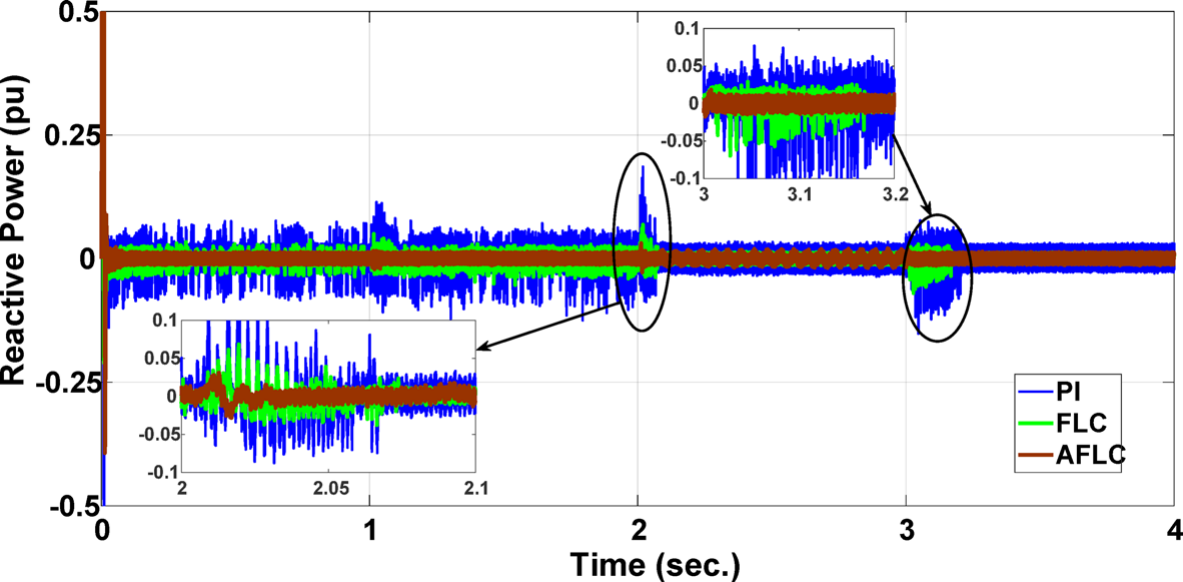
Reactive Power.

**Fig. 12:**
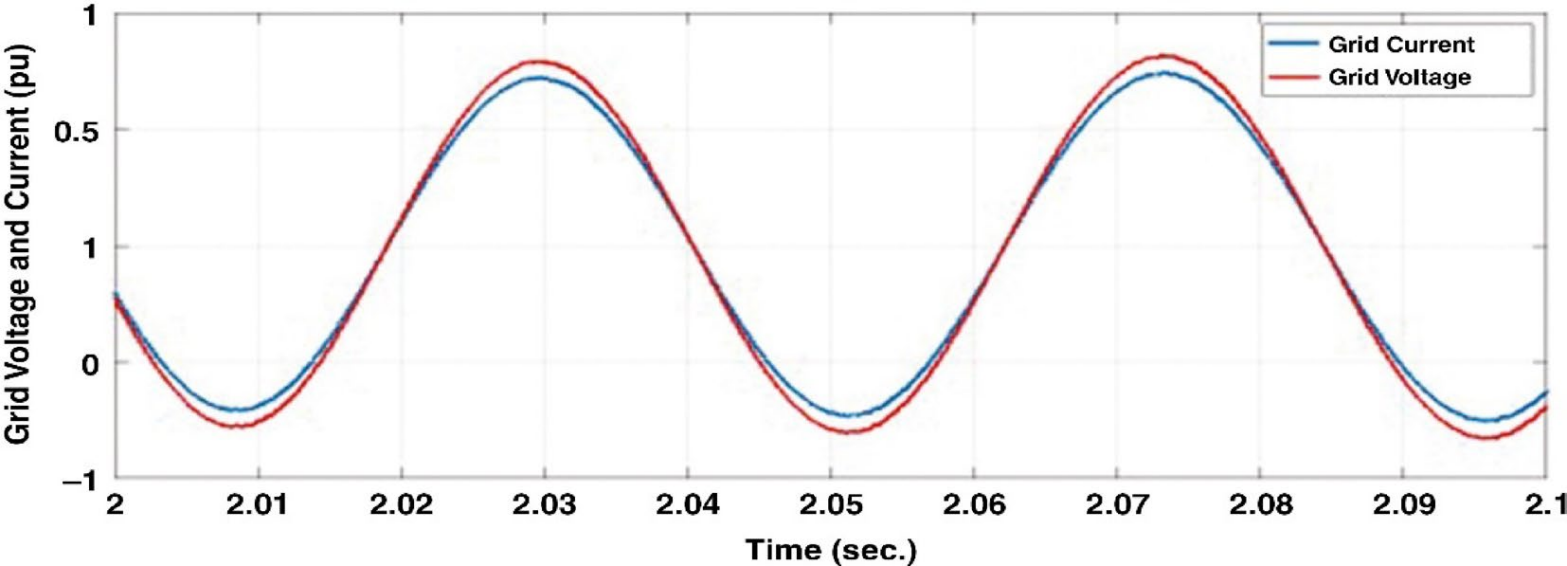
Grid current and voltage for the AFLC scheme.

**Fig. 13:**
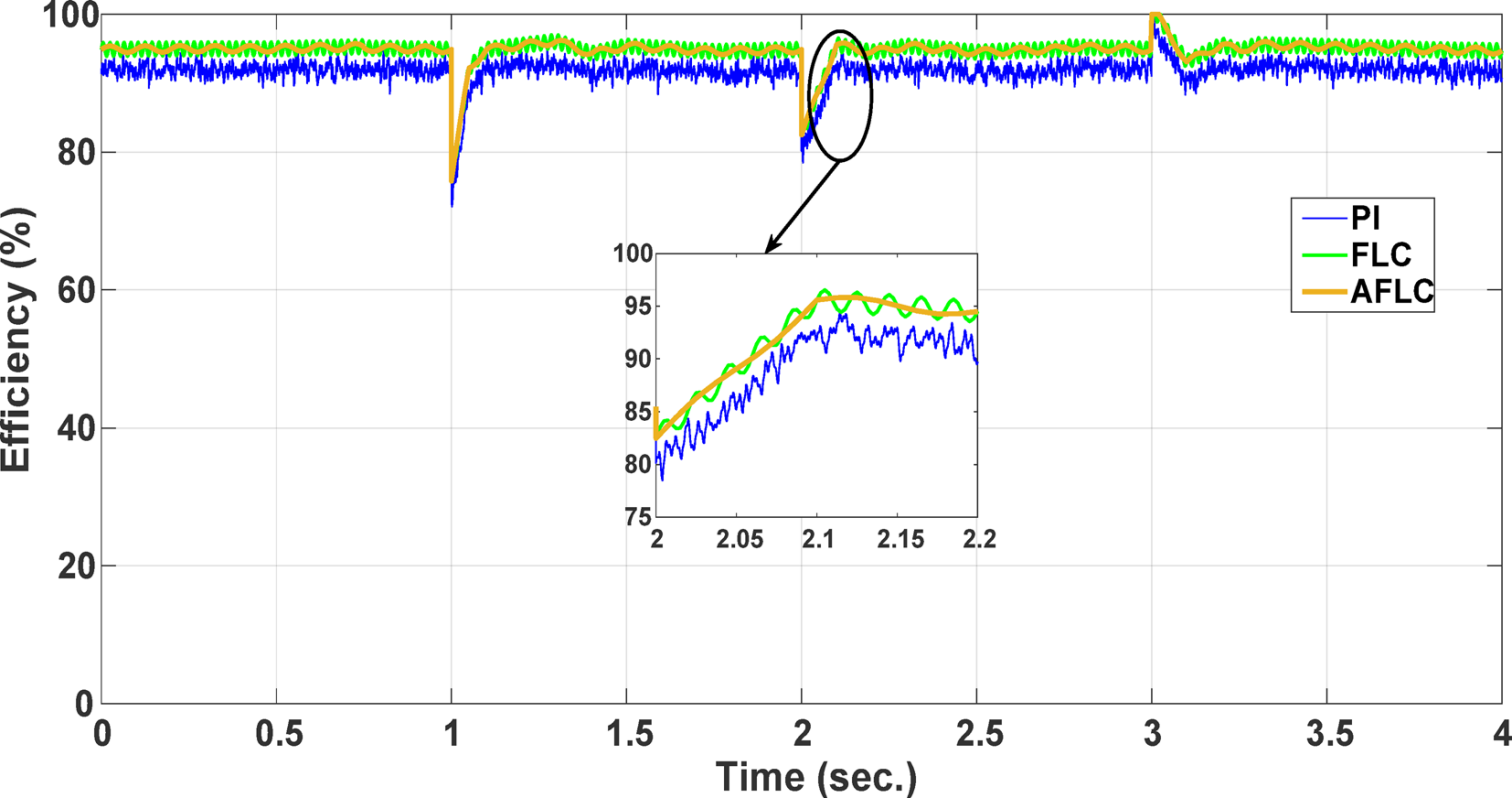
PV Generation System Efficiency.

**Fig. 14:**
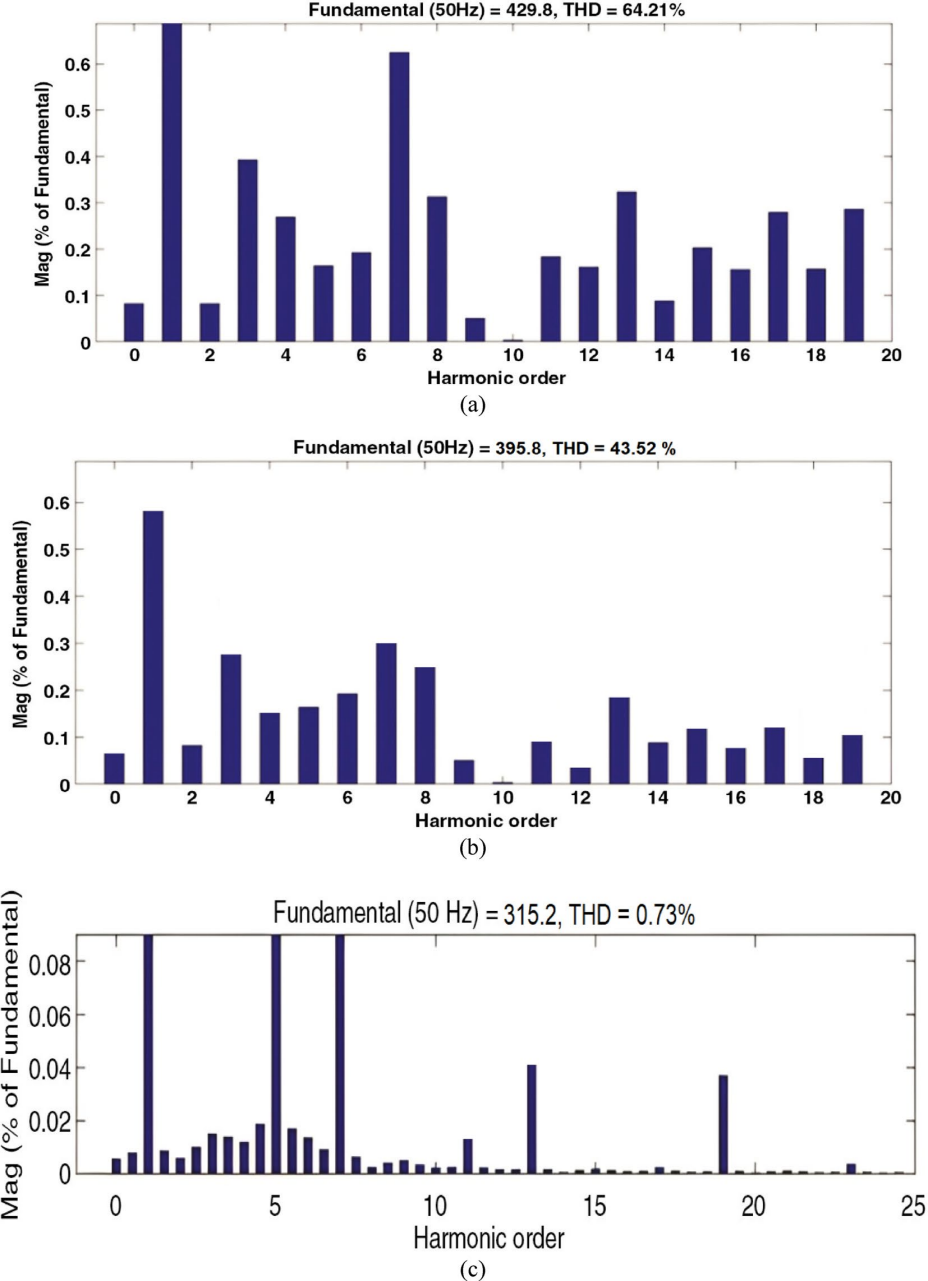
Total harmonic distortion of the inverter output current (**a**) PI, (**b**) FLC, and (**c**) AFLC.

This section evaluates the system’s behavior in response to stepwise variations in solar irradiance and the ambient temperature at 25 °C. The irradiance pattern, which alters over a 4-second duration, is presented in Fig. 7. The PV module characteristics confirm that the power output of the system varies with irradiance changes. The outcomes of this evaluation are detailed in the following discussion. Figure 8 shows the system’s power output. It can be observed that the amplitude of the power response changes in line with the irradiance profile. Compared to the FLC, the AFLC enhances power tracking performance by 68.26% and surpasses the standard PI controller by 86.25%. Furthermore, the AFLC displays better dynamic response and system stability, allowing quicker attainment of steady-state power. Table 3 shows the performance indices of output power. At t = 1.1s, the AFLC has the lowest peak overshoot compared to FLC and PI. At the time between 2 s and 3 s, AFLC minimizes the steady state error to minimum compared to other algorithms. The DC bus voltage (Vdc) behavior under varying environmental conditions is depicted in Fig. 9. Analysis reveals that all control techniques—PI, FLC, and AFLC—can regulate the DC voltage around the nominal 1pu value. Yet, the AFLC method exhibits superior performance, showing faster voltage settling and less chattering than both FLC and PI. Figure 10 presents the PV system current, demonstrating that AFLC more effectively tracks the desired reference current under environmental variability. This is due to minimized oscillations and higher system stability. The power delivered to the grid also shifts according to the variations in injected current. In Fig. 11, reactive power is regulated at zero to preserve power factor at unity. Among the three, AFLC and FLC exhibit the least error in reactive power regulation, with AFLC performing marginally better than FLC. These findings emphasize AFLC’s edge in maintaining accurate and stable operation during environmental changes, showcasing its effectiveness over traditional control strategies. In Fig. 12, the voltage and current of the grid are in phase, indicating the good reaction of the grid side converter controller when employing AFLC. Power factor operation is attained to unity as a result. Figure 13 illustrates the system efficiency with AFLC, FLC and conventional PI. The average value of efficiency during this period increased to 95.62% with AFLC scheme compared with 93.25% for the FLC scheme and 87.89% for the conventional PI scheme. In Fig. 14, The total distortion factor of the output current in case of PI is extremely high and reaches 64.21% compared with 43.52% for the FLC scheme and 0.73% for the AFLC scheme.

**Table 3:** The performance indices of output power.

	Time	PI	FLC	AFLC
**Rising time**	t = 1s	0.168s	0.112s	0.082s
	t = 2s	0.094s	0.082s	0.071s
**Peak Overshoot**	t = 1s	0.972pu	0.961pu	0.955pu
	t = 2s	1.492pu	1.455pu	1.421pu
**Settling Time**	t = 1s	0.283s	0.231s	0.175s
	t = 2s	0.422s	0.356s	0.231s

***Case II: Random Solar Irradiance***

**Fig. 15:**
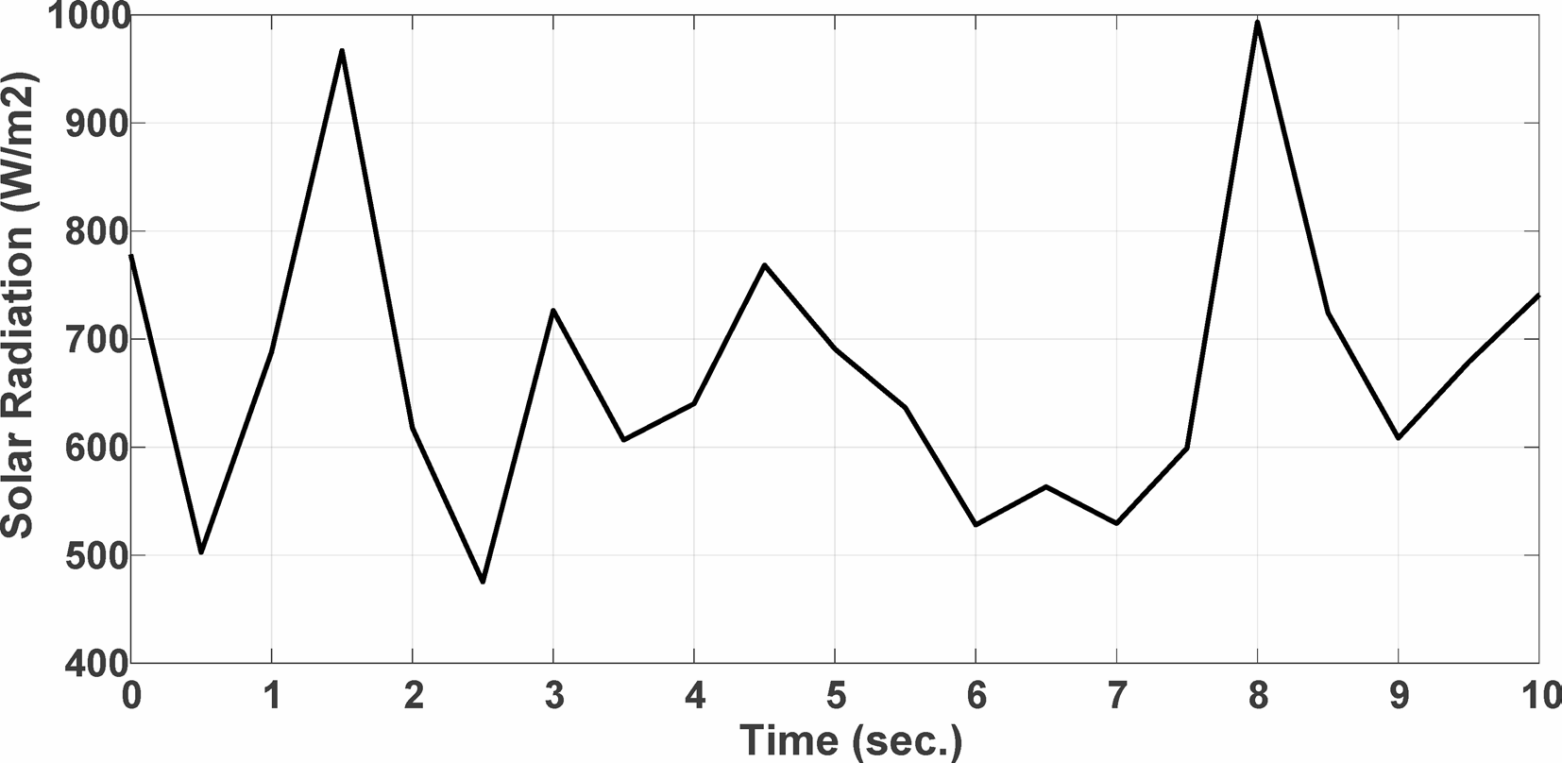
Solar Radiation.

**Fig. 16:**
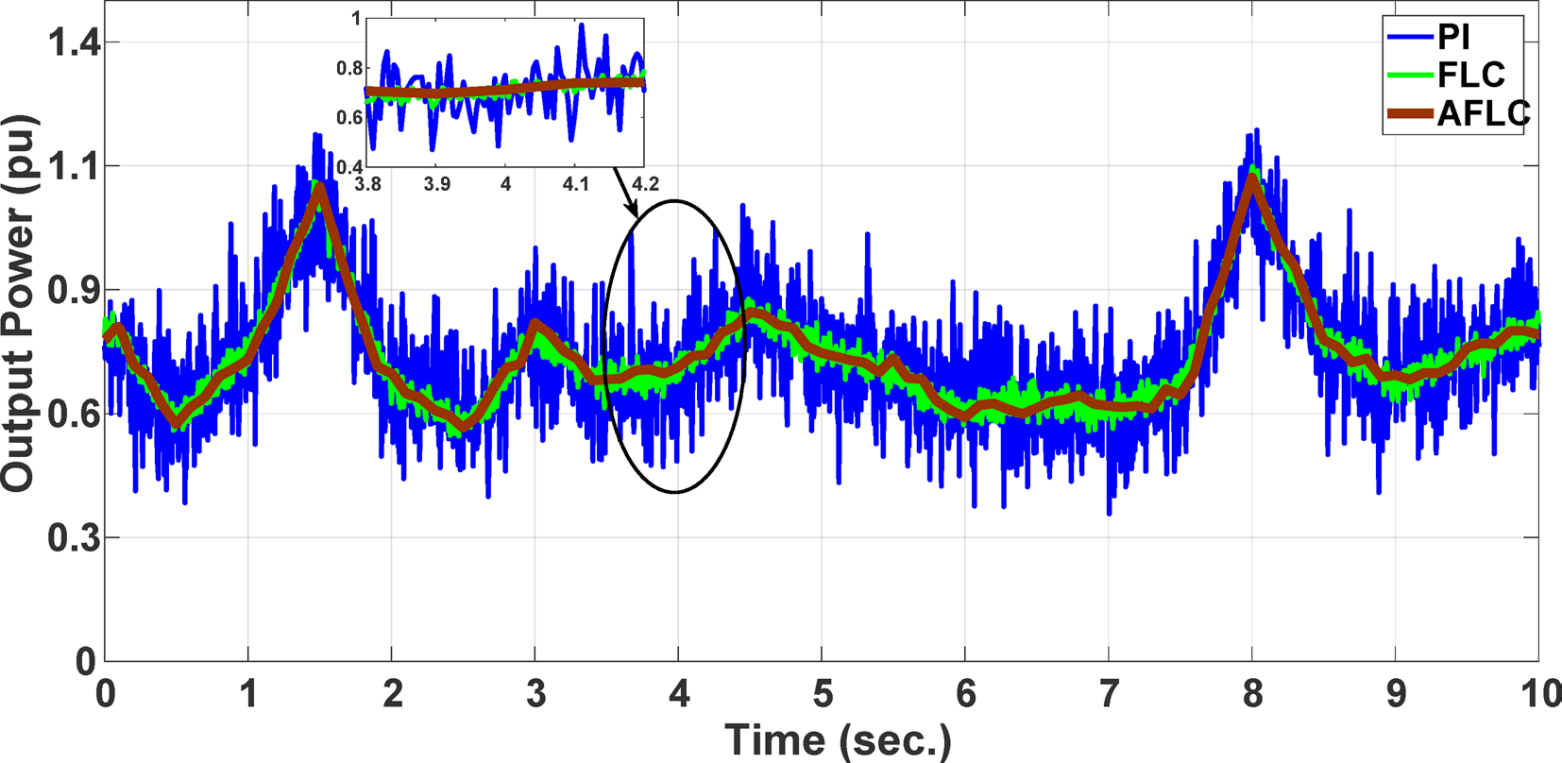
Output Power.

**Fig. 17:**
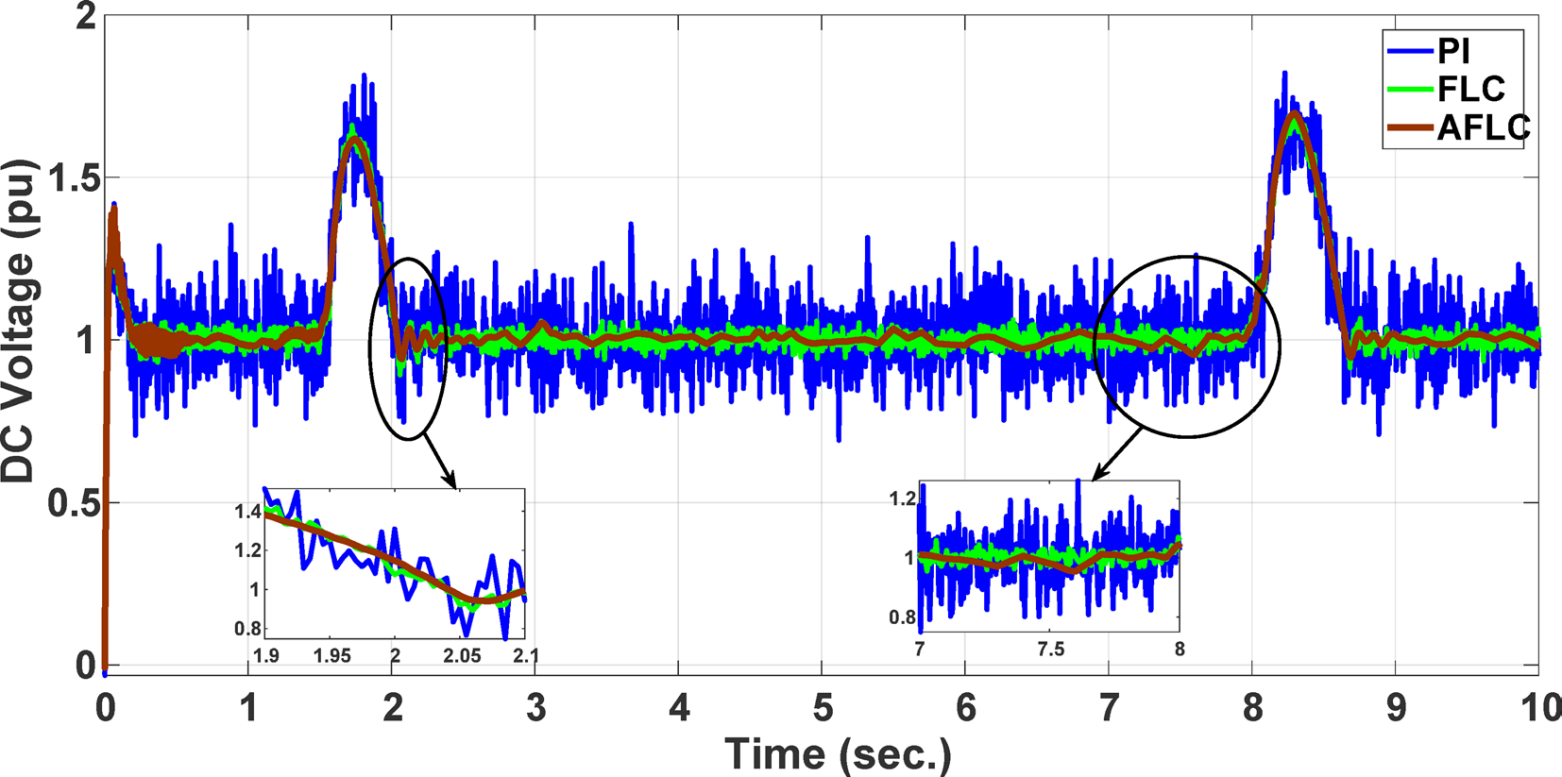
DC voltage.

**Fig. 18:**
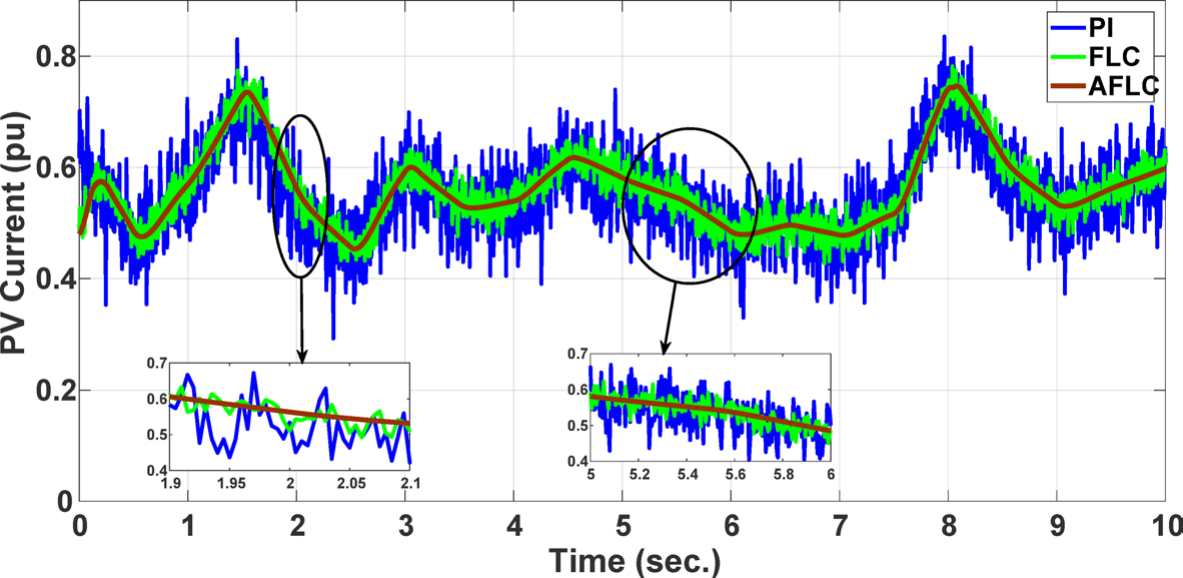
PV Current.

In the second scenario, the system is analyzed under random solar irradiance conditions. The performance of the AFLC is again benchmarked against FLC and PI controllers and evaluated. The solar irradiance profile, which changes in steps over a 10-second interval, is depicted in Fig. 15. The findings from this scenario are thoroughly analyzed below. Figure 16 presents the power out of the network. The amplitude of the power response fluctuates with changes in solar radiation. However, the AFLC technique shows greater stability, allowing power responses to achieve steady-state values more rapidly. At the time between 3.8s and 4.2s, AFLC has reduced the error to a minimum and improved the power tracking compared to other controllers. The DC bus voltage (Vdc) under dynamic climatic conditions is also examined, as shown in Fig. 17. The AFLC technique proves superior, offering quicker response time and less chattering compared to both the FLC and traditional PI controllers. The DC voltage error has reduced by 27.6% compared to PI and 12.32% compared to FLC. Additionally, Fig. 18 shows the PV current of the system, emphasizing that the AFLC technique excels over conventional controllers in tracking the reference current during varying climatic conditions. This improvement is due to its minimized oscillations and increased stability.

The AFLC exhibit enhanced characteristics that facilitate optimal tracking for power and restoration the performance of the system to a condition of steady state. Additionally, a compare between tracking errors is performed utilizing the Integral Absolute Error (IAE) to provide a more accurate assessment of the system’s efficiency:

65 $$\:IAE=\:{\int\:}_{0}^{\infty\:}\left|e\left(t\right)\right|dt$$

The errors for controllers of the PI, FLC, and AFLC, as detailed in Table 4, show a notable reduction. Specifically, the mean square error decreases by 79.67% in comparison to the PI controller and by 66.5% when compared to the FLC.

**Table 4:** The values of IAE Analysis.

	PI	FLC	AFLC
**Controller − 1**	0.112	0.064	0.013
**Controller − 2**	0.059	0.042	0.023
**Controller − 3**	0.153	0.087	0.003
**Controller − 4**	0.326	0.211	0.098
**Controller − 5**	0.0568	0.025	0.0067
***Mean square***	0.14136	0.0858	0.02874

### Experimental setup

**Fig. 19:**
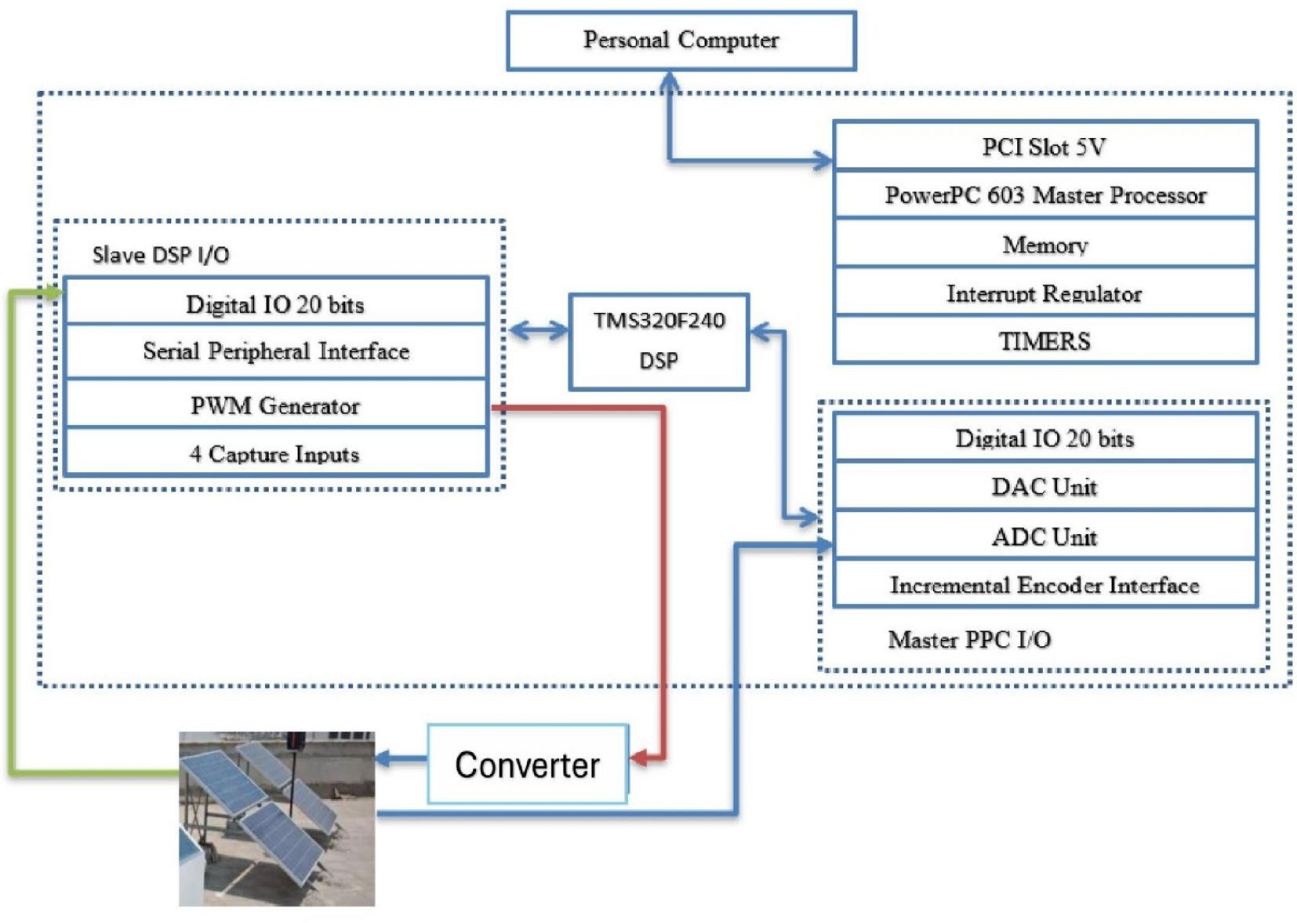
Schematic diagram of DSP 1104 controller card.

**Fig. 20:**
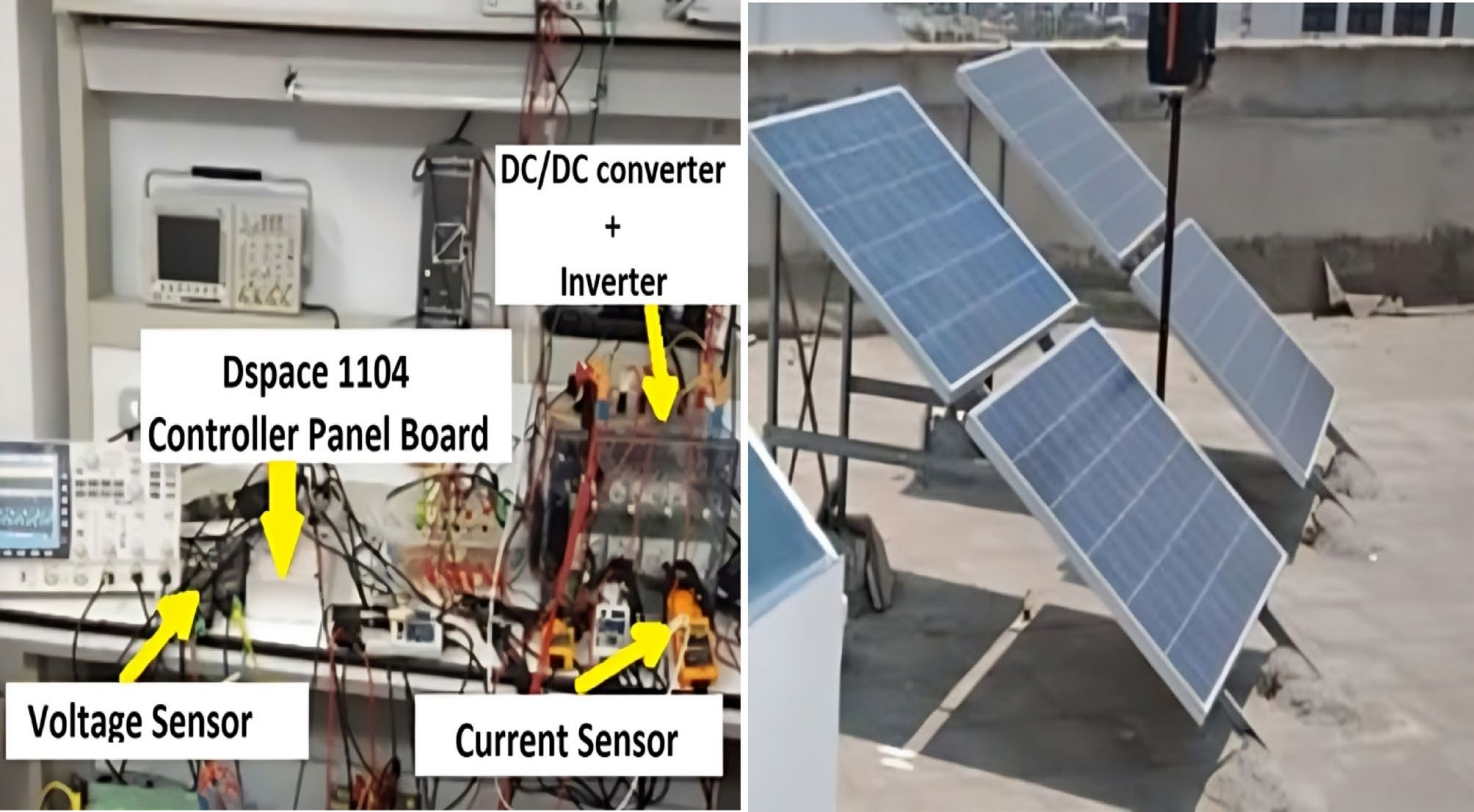
Hardware experiment.

To verify the results of the simulation and assess the feasibility of the controller which proposed in this study, the experimental hardware setup was built using a DS 1104 control board. Figure 19 illustrates the experimental configuration, which is based on a Digital Signal Processor DSP1104 control. Figure 20 displays the test of the real hardware for the setup of the laboratory. This system includes of 4 PV modules (FU240-250P, 305 W, V_mpp_ = 54.7 V, I_mpp_ = 5.58 A), The experimental setup includes a current measurement device and a DC-chopper circuit to control (featuring components such as an Insulated Gate Bipolar Transistor (IGBT) 600 V, 50 A, IR2110, 500 V offset, super-fast MUR 2060 rectifiers, and a 1N5819 diode), along with a three-phase inverter powered by a CM50DY-24 H MITSUBISHI module rated at 50 A/1200V.

**Fig. 21:**
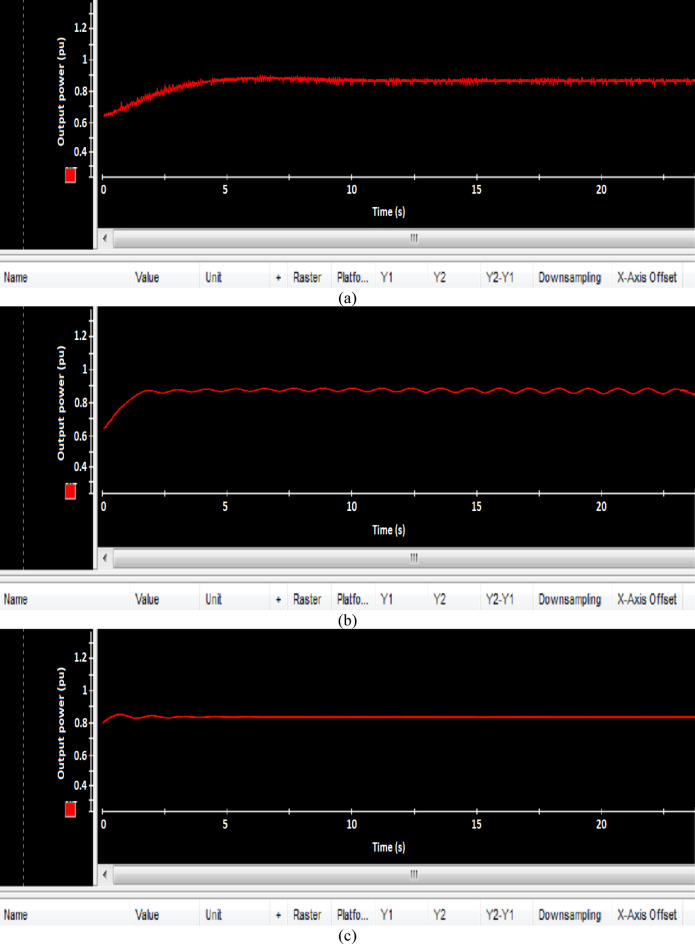
Experimental results of output power (**a**) PI, (**b**) FLC, and (**c**) AFLC.

**Fig. 22:**
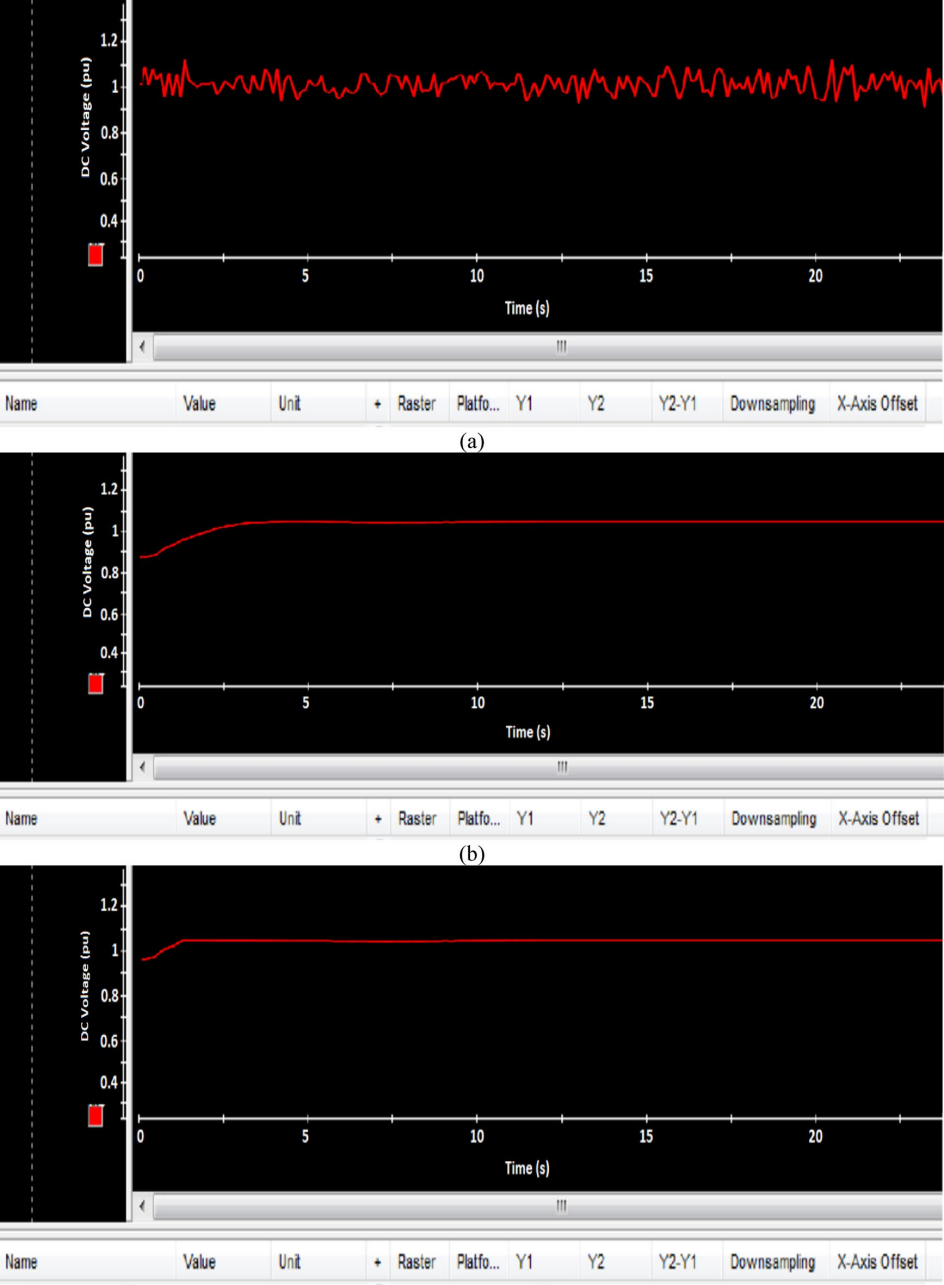
Experimental results of DC voltage (**a**) PI, (**b**) FLC, and (**c**) AFLC.

**Fig. 23:**
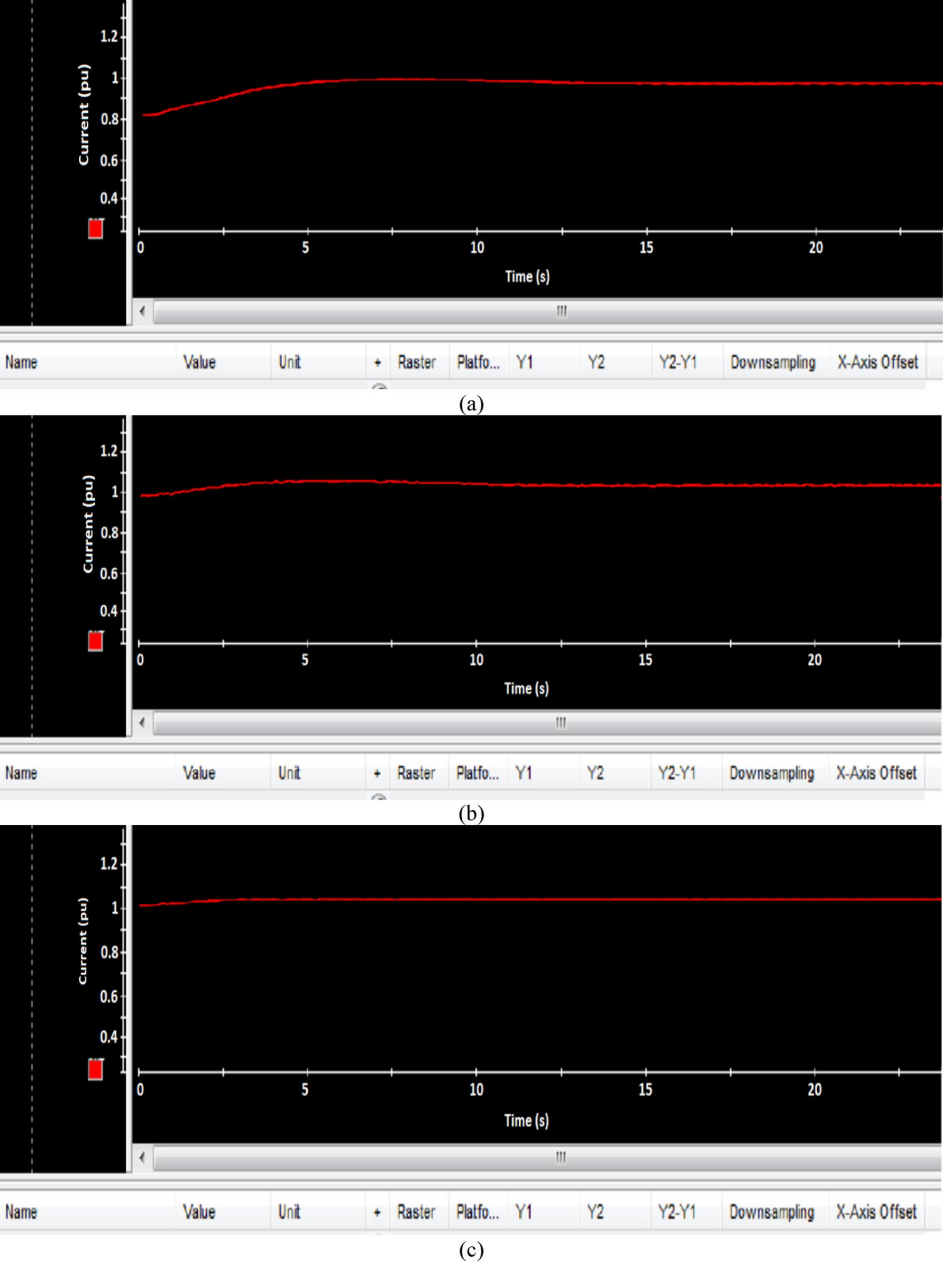
Experimental results of Current (**a**) PI, (**b**) FLC, and (**c**) AFLC.

### Experimental result

This section offers an in-depth analysis of the performance of the system under the given irradiance of 873 W/m² and a temperature of 29 °C. The output power of the photovoltaic (PV) system is significantly affected by solar irradiance, as illustrated by the PV module characteristics. In these conditions, the system’s behavior is evaluated using three distinct control strategies: PI, FLC, and the proposed AFLC. Figure 21 shows the power output of the network, which varies in amplitude based on fluctuations in solar irradiance. These variations directly affect the power response of the system. Notably, the AFLC (Adaptive Fuzzy Logic Controller) demonstrates superior performance by providing more stable power output. It allows the system to reach steady-state conditions more rapidly compared to the PI and FLC methods. Figure 22 illustrates the behavior of the DC bus voltage (Vdc) under changing environmental conditions. The three control strategies, FLC, and AFLC are all capable of regulating the DC voltage at the desired level of 1 pu. However, the AFLC technique clearly outperforms the others by exhibiting faster dynamic response and significantly reduced chattering. This suggests enhanced control quality and better voltage stability under variable irradiance and temperature conditions. Figure 23 presents the PV current behavior. The results show that the AFLC-based system has superior tracking performance, with minimal oscillations in comparison to the conventional FLC and PI controllers. This improved current tracking is essential for ensuring consistent power injection into the grid. As a result, the output power delivered to the grid also fluctuates more smoothly, reflecting the improved current control enabled by AFLC. Overall, the AFLC provides enhanced system stability and better adaptation to environmental changes.

### Conclusion and future work

This study demonstrated the effectiveness of the Adaptive Fuzzy Logic Controller (AFLC) in enhancing the performance, efficiency, and stability of grid-connected photovoltaic (PV) systems under varying irradiance conditions. The proposed AFLC was extensively evaluated through MATLAB/Simulink simulations and real-time experimental implementation using the DSPACE DS1104 control platform. The experimental results closely aligned with the simulation outcomes, confirming the accuracy and reliability of the controller under dynamic operating conditions.

The AFLC consistently outperformed conventional controllers, namely the Fuzzy Logic Controller (FLC) and Proportional-Integral (PI) controller, across several key performance metrics. It achieved faster response times, quicker convergence, reduced peak overshoot and undershoot, and significantly lower steady-state and mean square errors. Quantitatively, it improved power tracking by 68.26% over the FLC and by 86.25% over the PI controller. Additionally, the AFLC enhanced PV output power by 20% compared to FLC and 30% over PI, while reducing the mean square error by 79.67% compared to PI and 66.5% compared to FLC.

While the results confirm the AFLC’s high efficiency under dynamic irradiance, the controller has not yet been tested under more complex conditions such as partial shading or simultaneous changes in irradiance, temperature, and load. Future work will explore these scenarios and assess its practical deployment in larger-scale PV systems and diverse grid environments.

## Data Availability

The datasets used and/or analysed during the current study available from the corresponding author on reasonable request.

## Abbreviations

$$\alpha$$: Ideality factor
*V*_*oc*_: Open Circuit Voltage
$$\:{I}_{ph}\:$$: Photocurrent
$$\:{P}_{max}$$: Peak Power
$$\:{V}_{mp}$$: Rated Voltage
*I*_mp_: Rated Current
*R*_*s*_: Series resistance
*I*_*o*_: Saturation Current
*I*_*sc*_: Short Circuit Current
*T*_*a*_: Specific temperature
*R*_sh_: Shunt resistance
V: Output Voltage
I: Output Current
R: Load Resistance
*V*_⊥_: Thermal Resistance
P: Active Power
q: Reactive Power
*P*^*^: Desired active
*Q*^*^: Desired reactive power
ARC: Active Reactive Control
AARC: Average Active Reactive Control
PNSC: Positive and Negative Sequence Control
BPSC: Balanced Positive Sequence Control
PSO: Particle Swarm Optimization
ANN: Artificial Neural Network
IGBT: Insulated Gate Bipolar Transistor
